# Crystal Structure Prediction for Aprotic Ionic Liquids – Searching for the Unknown

**DOI:** 10.1021/acs.cgd.5c01674

**Published:** 2026-01-19

**Authors:** Petr Touš, Graeme M. Day, Ctirad Červinka

**Affiliations:** † Department of Physical Chemistry, University of Chemistry and Technology in Prague, Technická 5, Prague 6 CZ16628, Czechia; ‡ School of Chemistry and Chemical Engineering, 7423 University of Southampton, Southampton SO17 1BJ, U.K.

## Abstract

Ionic liquids (ILs) represent an extensively studied class of materials. Nevertheless, their solid state has often been overlooked, leading to frequent knowledge gaps about their phase behavior or crystal structures that such materials may form. This work focuses on the development of a crystal structure prediction (CSP) scheme suitable for aprotic ILs, relying on quasi-random crystal structure generation, dispersion-corrected density functional theory (DFT-D)-based energy reranking, and quasi-harmonic phonon treatment. The interpretation of peculiar differences in the crystallizability of very similar ILs upon cooling of their melts is presented. The versatility of the computational protocol is validated for [emIm]­[MeSO_3_], an IL known to be polymorphic. The current CSP identifies the [emIm]­[MeSO_3_] polymorph that is thermodynamically stable in reality at the top of the stability ranking, both in terms of DFT-D refined lattice energies and quasi-harmonic Gibbs free energies. Several low-energy, high-entropy crystal structures are also proposed for [emIm]­[MeSO_3_] as candidates for the remaining known polymorphs with yet unresolved crystal structures. Our CSP modeling explains the extraordinary reluctance of [emIm]­[EtSO_4_] to crystallize due to its glassy shape of the polymorph landscape with no distinct global energy minimum crystal structure.

## Graphical abstract

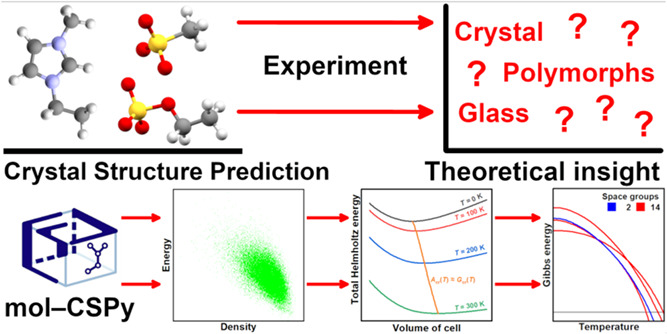

## 1 Introduction

Ionic liquids can be characterized as salts with particularly low melting temperatures – commonly required to range below 373 K.^1,2^ Most often, the cation of an IL is organic in nature and commonly features a system of conjugated double bonds, being beneficial for sufficient charge delocalization, or a quaternary cationic site. In turn, the anion can be either organic or inorganic, in both cases exhibiting a sufficient electronegativity to stabilize its negative charge.

ILs have potential applications throughout chemical technologies, as solvents with a potentially low environmental footprint. This is due to the fact that their vapor pressures are extremely low,^2^ and thus ILs would not leak via atmosphere when used on large scale. However, any hopes for applications require sufficient exploration, and possibly also optimization of their properties and phase behavior – their extremely low vapor pressures are a prominent example, as before the discovery of this phenomenon it was postulated that ILs do not have any vapor pressure at all.

Over the last decades, the literature exploring properties of ILs has grown immensely wide.^3,4^ Most of this literature naturally focuses predominantly on the liquid state of ILs. Although there are some detailed experimental investigations of the phase behavior of ILs including determinations of their crystal structures,^5–17^ such studies are rather scarce in the global IL context. In particular, there are several studies aiming at exploring the phase behavior of aprotic ILs including their solid state, and at thermodynamic assessment of their polymorphism.^5,6^

It has to be mentioned that the crystallization of ILs may be difficult in general. Strong Coulombic interactions impart a cage effect that restricts ionic diffusion in the bulk that may in turn hinder molecular rearrangements to a crystal structure upon cooling.^5,6^ ILs are thus prone to massive undercooling of their liquid phase, leading to deeply supercooled metastable liquids that can be stored even dozens of degrees Kelvin below their equilibrium melting temperatures. Such supercooled liquids typically undergo vitrification, i.e., formation of an amorphous solid phase upon further cooling rather than a proper crystallization.^6^ Another problem with experimental phase behavior studies of ILs is that they are extremely sensitive to water content,^6^ and thus have to be repeatedly dried, lest the observed phase behavior can be subject to various ambiguities or controversies.

One of the archetypal organic cations used to design ILs is the ethylmethylimidazolium ([emIm]) species. Certain recent experimental studies, combining calorimetry and X-ray diffraction, revealed that even ILs coupling [emIm] with very similar anions behave extremely differently in terms of their crystallization. For example, ethylmethylimidazolium methanesulfonate ([emIm]­[MeSO_3_]), while crystallizing rarely, does form four different polymorphs with only the crystal structure of the most stable polymorph being currently known.^6^ These experimentally measured transition temperatures and enthalpies of the known polymorhps of this IL are given in Table 1. On the other hand, ethylmethylimidazolium ethyl sulfate ([emIm]­[EtSO_4_]) did not crystallize in any of the reported extensive experiments, instead always forming only a glassy solid phase upon cooling (even slow) of its melt^6^ with a reported glass transition temperature of *T*_glass_ = 189.4 K allowing for some variation depending on the applied heating rate.

**Table 1:** Experimental Descriptors of the Solid–Liquid Equilibrium Determined at *p* = (100 ± 10) kPa in Ref 6

polymorph	enthalpy of fusion (kJ mol^–1^)	melting temperature (K)
crI	15.8 ± 0.4	311.3 ± 0.3
crII	21.1 ± 0.5	309.5 ± 0.3
crIII	17.0 ± 0.7	308.4 ± 0.7
crIV	20.6 ± 0.8	292.3 ± 0.7

Another recent study included molecular dynamics simulations of structural, energetic, and transport properties of the liquid phases of various ILs including [emIm]­[MeSO_3_] and [emIm]­[EtSO_4_].^18^ Having compared mean coordination numbers in bulk liquid, typical interaction energies of the counterions, cohesive energy density, ionic self-diffusivities and similar parameters for the apparently noncrystallizing [emIm]­[EtSO_4_] with other ILs capable of routine crystallization, that study revealed that there are no particular reasons why the [emIm]­[EtSO_4_] would not be able to crystallize and why the other, for instance [emIm]­[MeSO_3_] would.^18^

In this work, a different computational perspective is adopted to interpret the previously observed differences in crystallizability of the mentioned ILs using modern CSP methods. We aim at predictions of what could be the crystal structures of the experimentally observed, yet unresolved polymorphs of [emIm]­[MeSO_3_], and also whether there are any potential stable crystal structures that could accommodate the packing of [emIm]­[EtSO_4_]. Since the considered ions exhibit some elementary torsional flexibility, a question about what could be the possible molecular conformations of those compounds needs to be solved within such in silico CSP. Finally, the task of modeling the relative stability of predicted crystal structures is required, imparting the need for developing a reliable, efficient and high-throughput computational methodology.

The inability of crystal engineers to predict crystal structures of even the simplest compounds used to be a controversial subject several decades ago.^19^ Since then, tremendous progress has been made within this field, as is evidenced by the ongoing Crystal Structure Prediction Blind Tests, regularly organized by the CCDC, with the results of the latest test being published in 2024,^20^ including some success stories even for relatively flexible target systems. Our intention therefore was to develop a validated methodology to predict crystal structures of ILs to reproduce the known phase behavior of [emIm]­[MeSO_3_], and then use this verified methodology to predict putative structures of [emIm]­[EtSO_4_] and gauge their stability using known first-principles methods of thermodynamic modeling. The molecules making up these two target systems can be found in Figure 1.

**Figure 1:**
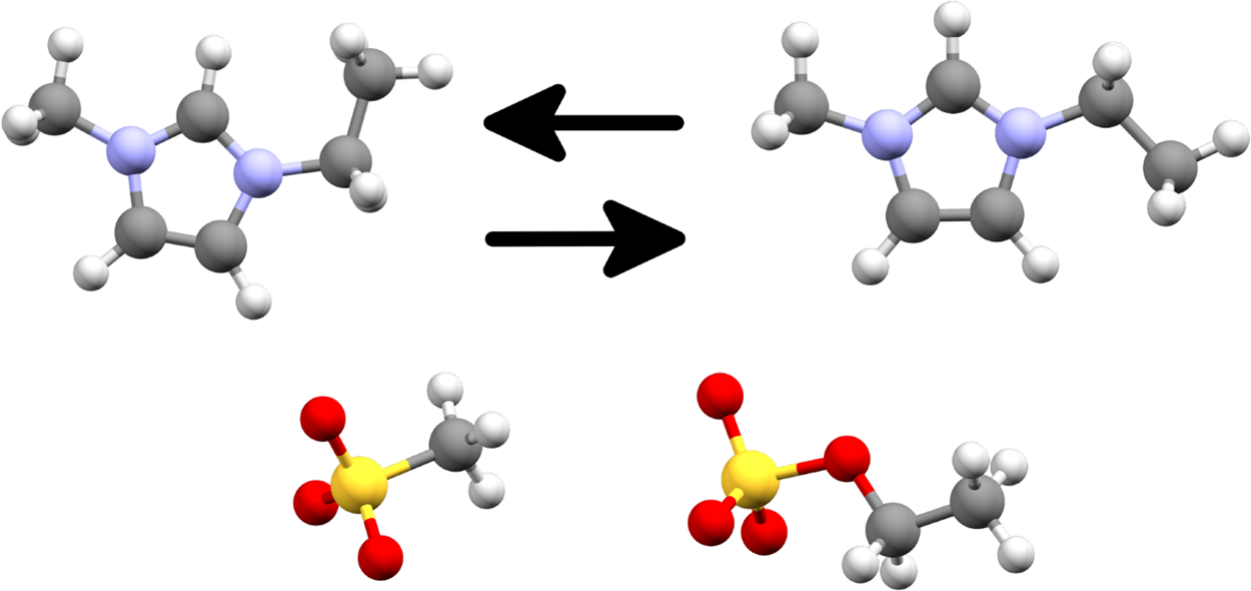
Overview of ions constituting the target ionic liquids. The top row represents the two possible conformations of the cation, which is the same for both of the ionic liquids. On the left is the conformation found in the experimental crystal structure of [emIm]­[MeSO_3_], which we will label as Conformation 1 in the rest of the text, whereas on the right is the second conformation we considered for the purposes of a thorough structural search, which we will label as Conformation 2 in the rest of the text. The bottom row represents the two anions which constitute the two different ionic liquids – left: methanesulfonate, right: ethyl sulfate.

## 2 Computational Methodology

The task of CSP is often divided into two distinct sections, the first being structure generation and the second being structure reranking.^21^ Either one can be set up in a myriad of ways, allowing for flexibility and adaptability to a particular target system. Given the virtually limitless possibilities for molecules to be packed within a crystal lattice and, by contrast, limited time and computational resources, the reoccurring theme in any CSP scheme is sieving through structures using several steps of selected methodologies, increasing the level of methodology in subsequent steps, so that computationally demanding calculations are applied to the most promising structures. This is often referred to as a “funnel” of sorts. An overview of the methodology we used is shown in Figure 2.

**Figure 2:**
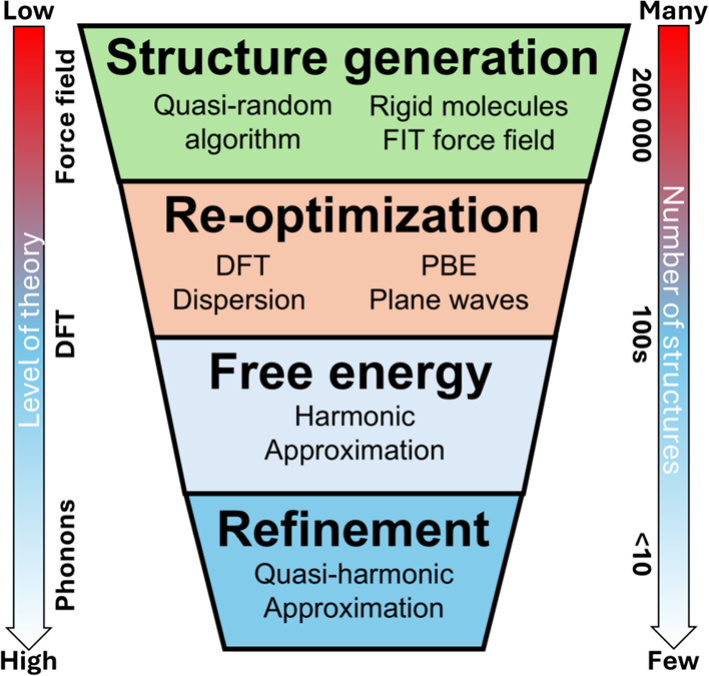
A simplified representation of the crystal structure prediction methodology used in this work. During the course of the steps on this diagram, a decreasing number of structures is being treated at an increasing level of theory – this is often stylized as a “funnel”.

### 2.1 Structure Generation Stage

In the structure generation stage, we assumed that all studied molecules are mostly rigid. For all predicted crystal structures, we assumed that the ethyl moieties in the [emIm] cation and in the [EtSO_4_] anion attain a conformation extended (see Figure 1) away from the molecular core, as any other conformation would require an unachievable stabilization from the crystal packing to be stable. We assumed a single conformation of the [EtSO_4_] molecule to be considered, owing to the symmetry of the SO_3_ moiety relative to the EtO group. For [emIm], we assumed two unique orientations of the ethyl group, which were treated separately – each conformation resulted in the −CH_3_ moiety lying in the plane defined by the central aromatic ring of [emIm]. We decided this based on the fact that the two conformations differ by only about 2.7 kJ mol^–1^ in the gaseous phase. We label these as *Conformation 1* and *Conformation 2* in the rest of the text (see Figure 1).

The initial stage was based on quasi-random generation of trial crystal structures, as implemented in the program mol-CSPy.^22,23^ In essence, the program uses a low discrepancy, quasi-random sequence to generate lattice parameters, positions of the rigid molecules and their relative molecular orientations within the unit cell, based on a specified space group. These generated structures are screened for nonphysical positions, such as overlapping molecules; molecular clashes are relieved through unit cell expansion up to a volume limit and structures are discarded if clashes cannot be removed. After this process is complete, a quasi-Newton–Raphson optimization is performed to minimize the lattice energy. Its repulsion-dispersion contribution is expressed as a sum of pairwise anisotropic molecular Buckingham potentials based on the FIT force field.^24–27^ Electrostatic interactions are modeled via atom-centered multipoles up to rank 4, i.e., hexadecapoles with values derived from a distributed multipole analysis^28^ (calculated separately for each molecular ion, in each conformation). This level of theory should be accurate enough to filter out obviously unfavorable structures while being sufficiently efficient to compute in a high-throughput regime.

Only selected space groups were selected for the structural generation: *P*1̅ (2), *P*2_1_ (4), *C*2 (5), *Cc* (9), *P*2_1_/*c* (14), *C*2/*c* (15), *P*2_1_2_1_2_1_ (19), *Pca*2_1_ (29), *Pna*2_1_ (33) and *Pbca* (61), as the vast majority of known crystals found in the Crystal Structure Database (CSD)^29^ exist within these space groups.^30^ It is recommended that the number of structures generated this way be in the order of tens of thousands for each considered space group, so that adequate screening of the vast configurational space is achieved. In this case, we chose to generate structures in each of the ten space groups until 10,000 structures per space group were successfully energy-minimized. Given the fact that we considered each [emIm] conformation as separate molecules, and assuming only the *Z*′ = 1 case, this gave us in total 200,000 generated and energy minimized trial structures in the searches for each of the two compounds. After the search was completed, the obtained set of structures was consolidated (for each [emIm] conformation separately) and checked for structure duplicates using the tools provided in mol-CSPy: fast comparison of simulated powder X-ray diffraction patterns, followed by comparison using the COMPACK algorithm.^31^ Total relative energies were evaluated by combining the force field intermolecular energies with the intramolecular conformational energy difference.

The energy model provided by the force field served as an initial assessment of the structure ranking. From each of the obtained sets of structures (two cation conformations for each compound), we selected the lowest 10 kJ mol^–1^ window of structures for the purposes of reranking, as these should contain the most promising crystal structures.

### 2.2 Structure Reranking Stage

As was mentioned in the previous section, all structures within a certain energy window from the force field based predictions were reranked using higher level calculations. While high quality force field methods can provide reliable CSP,^32^ it has been long established that methods of quantum chemistry offer greater reliability, in particular for molecules with conformational flexibility, with dispersion-corrected density functional theory (DFT-D) being the leading method of choice for molecular crystals.^21^ This is crucial, as computational exploration of known polymorphic compounds has established that the differences between individual polymorphs rarely exceed 10 kJ mol^–1^ and predominantly lie within 2 kJ mol^–1^ or even less, i.e., subchemical accuracy in terms of predicted lattice energies is due in the structure reranking context.^33,34^ Aiming at polymorph ranking at finite temperatures, the ranking process ought to include the treatment of crystal phonon modes, which govern any variation of the ranking with respect to temperature, contributing non-negligibly to relative free energies as temperature increases.^35^

Unless specified otherwise, all calculations utilized the PBE functional,^36^ with the basis set functions taking the form of plane waves in the projector-augmented wave (PAW) formalism.^37,38^ Furthermore, the nonvalence electron shells were treated by PAW potentials. For the dispersion correction, we chose the semiempirical Grimme dispersion correction (D3) in combination with the Becke–Johnson (BJ) damping function, or D3­(BJ).^39,40^ These calculations were performed in the computer program VASP, version 5.4.4.^38,41–44^ Unless stated otherwise, the calculations assumed a full relaxation of both cell parameters and atomic positions. We will be referring to this setup as PBE hereafter. The total overview of the computational methodology in the reranking stage is summarized in Table 2.

**Table 2:** A Summary of the Computational Methods Used in the Structure Reoptimization Stage^a^

stage	PAW potential	PAW cutoff (eV)	ranking criterion	*N*
initial reoptimization	regular	500	*E*_PBE_^el^(*V*_0_)	∼200–250
harmonic treatment	regular	900	*G*_PBE_^HA^(*T*)	∼15–20
*E*(*V*) curve calculation	hard	900	*E*_PBE_^el^(*V*)	∼15–20
quasi-harmonic treatment	hard	900	*G*_PBE_^QHA^(*T*)	<10
B3LYP correction	hard	900	*G*_PBE_^QHA^(*T*) + Δ*E*_B3LYP_	<10

a All calculations of the electronic energy *E* ^el^ and harmonic or quasi-harmonic Gibbs energy *G* use the PBE-D3­(BJ)/PAW level of theory unless noted otherwise. The value *N* relates to the number of structures treated for each compound in total.

The initial reoptimization step provided us with an idea of the shape of the polymorph landscape for each compound. This allowed us perform the first shortlisting, retaining promising structures from each of the sets to be treated further – for each of the unique [emIm] conformations, we selected about ten structures, trying to strike a balance between the computational cost of the proceeding calculations and the inclusion of all the important structures. As an initial method of refinement, we chose to calculate the vibrational frequencies (phonons) of the generated structures after the optimization via the harmonic approximation. This calculation was based on treatment of supercells exceeding 10 Å in each dimension^45^ and on the finite displacement method as implemented in the program Phonopy,^46,47^ which was also used to evaluate the thermodynamic contribution to the Gibbs free energy stemming from the harmonic phonon modes, including its dependence on temperature.

Owing to the fact that the phase behavior of [emIm]­[MeSO_3_] has been studied experimentally previously,^6^ we wanted to achieve the highest possible computational accuracy, and to validate our in silico thermodynamic assessment of our predicted candidate structures by comparison with the available experimental data. While the harmonic approximation does provide us with the necessary Helmholtz or Gibbs energies, it neglects crucial effects that influence the thermodynamic behavior of crystals, most notably the thermal expansion. Its omission may have an adverse effect on the computational accuracy of the polymorph ranking, especially at elevated temperatures.

The quasi-harmonic approximation (QHA),^45,48^ which we employed for selected structures that passed through another shortlisting step, attempts to address these shortcomings. Within the QHA, the Helmholtz energy is evaluated as per thermodynamic relations, i.e., as a function of *both* temperature and volume.

The QHA aims to model how the static (electronic energy *E*_el_) and dynamic (phonon frequencies) descriptors of the crystal vary with respect to its volume. To access the static ingredients of QHA, one performs a series of unit-cell optimizations at a constant volume – that is compressing/expanding the optimized crystal structure by some factor and optimizing the cell parameters and atom positions anisotropically while keeping the unit-cell volume constant. The resulting dependence of energy on volume is often referred to as the *E*(*V*) curve, and in the case of this paper, we chose to compute the *E*(*V*) curves over volume factors from *V* = 0.98*V*_0_ to *V* = 1.08*V*_0_ with a step in the scale factor of 0.01, where *V*_0_ is the unit-cell volume after the first optimization (allowing the cell volume to relax). Following this evaluation, the *E*(*V*) curve was fitted to the Murnaghan equation of state.^45,49^

The practical implementation of the dynamic QHA ingredients consists of phonon calculations at several crystal volumes near the *E*_el_ minimum. Thence obtained vibrational Helmholtz energy *A*_vib_ is interpolated as a function of crystal volume at individual temperatures, yielding a two-variable function *A*_vib_(*T*, *V*). Adding the static and dynamic components together yields the total Helmholtz energy *A*(*T*, *V*) = *E*_el_(*V*) + *A*_vib_(*T*, *V*) of the crystal as a function of temperature and volume. For the purposes of structure ranking, we used the equilibrium Helmholtz energy, that is the minimal Helmholtz energy at each temperature, which corresponds to the equilibrium volume at each temperature from the *A*(*T*, *V*). Assuming *p* = 0, the Helmholtz energy is equal to the Gibbs energy – for the sake of convenience, we will refer to the thermodynamic property resulting from the QHA and the ranking criterion used to compare structures as the Gibbs energy. This assumption is valid when attempting to rank at ambient pressures, as the error introduced by omitting the *pV* term is negligible for such a low value of pressure in comparison to other errors introduced by the QHA or the underlying GGA-tier of the DFT functional.

As a final point of accuracy improvement, we employed a correction to a more accurate and sophisticated hybrid DFT functional, namely the B3LYP functional. The calculations in the solid phase are often not performed using the B3LYP functional due to the sheer computational cost, and the impractical manipulation of the exact exchange terms within the PAW framework. These practical obstacles manifest particularly when performing structure optimizations. In order to perform this correction, we simply calculated the single-point B3LYP-D3­(BJ)/PAW (we will refer to this setup as B3LYP henceforth) energy for the crystal structure corresponding to the minimum on the *E*(*V*) curve and computing the difference Δ*E*_B3LYP_ = *E*(*V*_0_)_B3LYP_ – *E*(*V*_0_)_PBE_.^50,51^

## 3 Results and Discussion

### 3.1 Crystal Structure Prediction for [emIm]­[MeSO_3_]

According to the methodologies described in Section 2, we attempted to computationally reproduce the known experimental crystal structure of [emIm]­[MeSO_3_], as well as to assign crystal structures to the other polymorphs observed in previous phase behavior studies for this material. Following the successful generation of two hundred thousand crystal structures (one hundred thousand for each unique [emIm] conformation) of [emIm]­[MeSO_3_], the structures were checked for duplicates as described in Section 2.1, resulting in about 35 000 unique structures covering a lattice energy range of approximately 90 kJ mol^–1^. The entire polymorph landscape is shown in Figure 4 (left), where the energy ranking is based on the FIT force field energy. Since we wanted to reproduce the truly existing polymorph in our structure generation, we first checked whether this polymorph landscape contained the known experimental structure of [emIm]­[MeSO_3_] (CSD^29^ REFCODE SAGPOJ).^6^ The experimental structure has been found by the structure generation procedure; however, its position according to the FIT force-field ranking does not correspond to the most stable structure, but it resides within the 10 kJ mol^–1^ window of the best structures.

We found that not only was mol-CSPy able to generate the known experimental structure, the force field energy ranking also placed it in a reasonable energy window from the lowest energy structure, as can be seen at the very bottom of Figure 4 (left). Although the experimental structure was not placed decisively at the very best position in the landscape (sufficiently high density and the lowest energy), this is still an encouraging result, since neither the force field nor the mol-CSPy tool were specifically designed for predicting crystal structures of ionic liquids. Comparison between the experimental structure and the mol-CSPy generated structure can be found in Figure 3.

**Figure 3:**
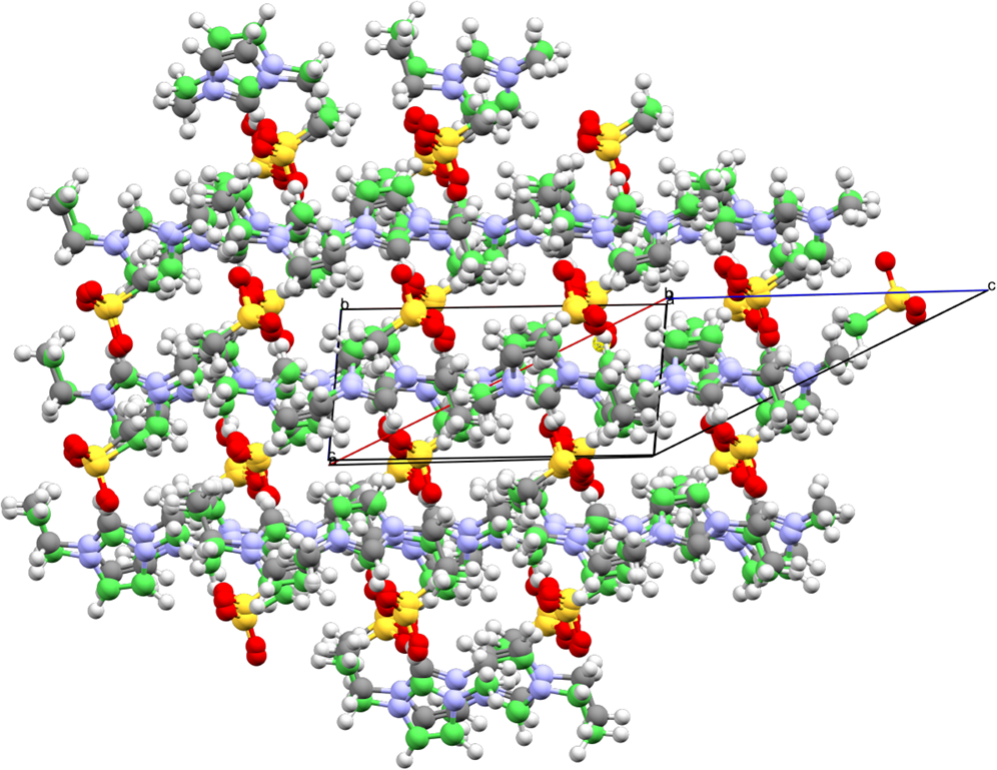
Comparison between the experimental structure (natural colors) and the structure generated via the mol-CSPy program (green), i.e., it was ranked based on the force field energies. According to this ranking, the generated structure lies Δ*E*_FF_ = 7.304 kJ mol^–1^ above the global minimum. A total of 70 molecules from both lattices are being compared in this figure – the color green denotes that all molecules from the generated structure passed the comparison.

In accordance with the methodology described in Section 2.2, we took the lowest 10 kJ mol^–1^ of structures (filled-in symbols in Figure 4 (left)) generated this way and subjected them to an optimization in accordance with the level of theory specified in the first row of Table 2. The results of this optimization are presented in Figure 4 (middle).

**Figure 4:**
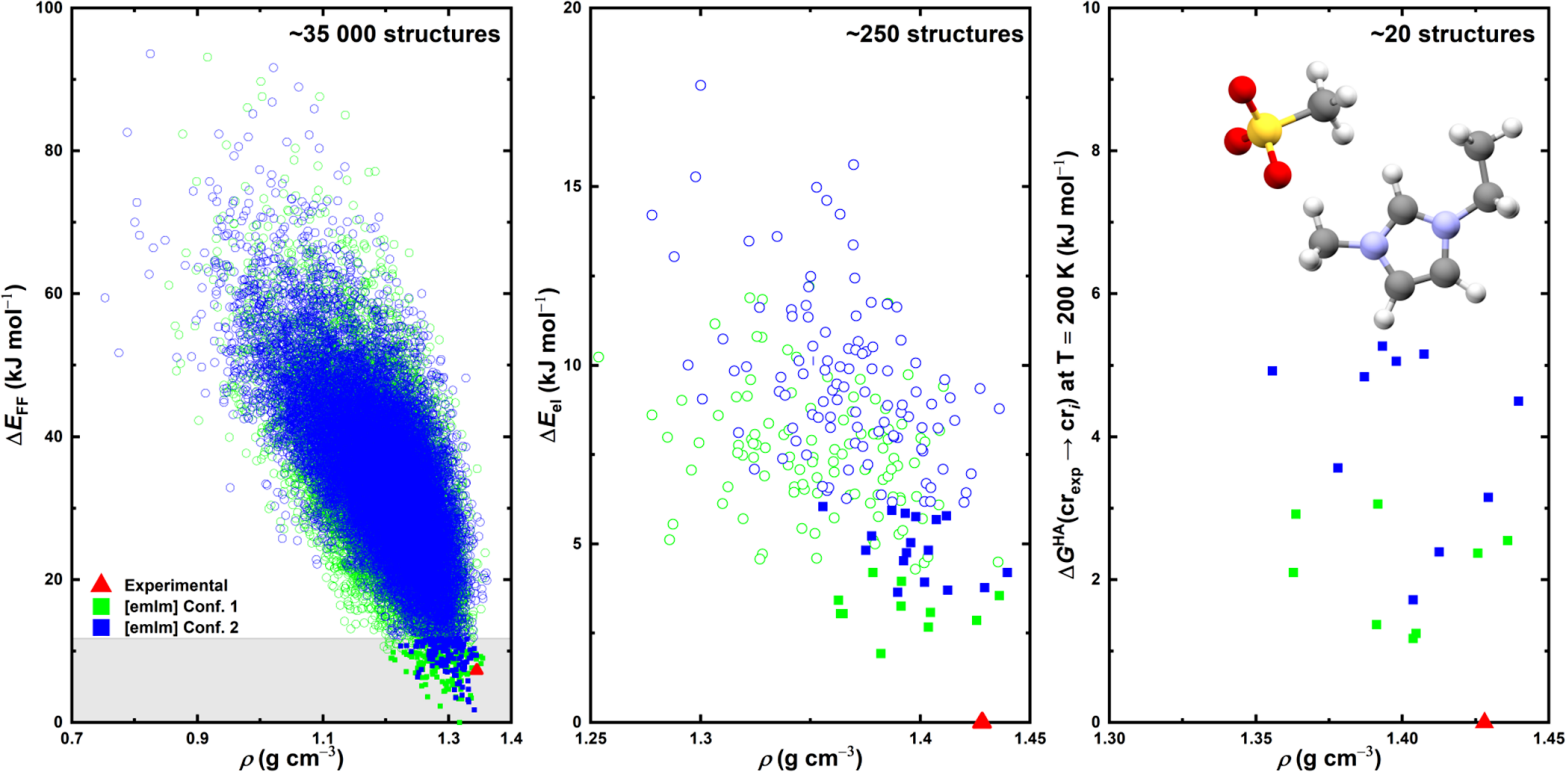
Polymorph landscapes predicted in the CSP process for [emIm]­[MeSO_3_] illustrating the shortlisting of potential structures throughout the CSP funnel scheme. Left: The global [emIm]­[MeSO_3_] landscape generated using the mol-CSPy program with relative energies Δ*E*_FF_ corresponding to the FIT force field. The gray bar helps denote the energy of the last structure considered in further calculations. Middle: The landscape of the selected [emIm]­[MeSO_3_] structures from the figure on the left (full squares) when put through a PBE-D3­(BJ)/PAW reoptimization. Right: The Gibbs energy computed in the harmonic approximation at *T* = 200 K for selected structures (full squares) from the figure in the middle. Hollow points in all boxes denote structures that were discarded from further modeling after the particular calculation steps, while solid squares denote structures that were selected for further analysis in subsequent steps. In all figures, the red triangle denotes the position of the experimental structure found by the initial algorithm run.

An empirically parametrized force field such as FIT is not expected to provide accurate rankings for systems that are far from those for which it was designed: here, net molecular charges, molecular flexibility and elements that were not in the original force field parametrization. Therefore, in the CSP context, the force field’s role is to provide fast optimization of trial crystal structures and to filter out obviously incorrect structures. Since the given FIT force field does not explicitly assume atomic polarizability and it does not reflect the quantum nature of electrons in the bulk crystals, one should expect some flaws of its description of bulk energies. Comparing the relative energies modeled by the FIT force field and by the PBE-D3 theory, we found that among roughly 500 low-energy structures, there is a root-mean-squared deviation (RMSD) of about 4.6 kJ mol^–1^ which is an important value guiding one to set the threshold for retaining predicted structures throughout the CSP funnel workflow. Similarly, we found that PBE-D3 optimization led to additional 5.1 kJ mol^–1^ RMSD in relative energies within that subset of structures. Quantification of these RMSD values is important to verify that inconsistencies between the force field and QM methods does not play an important role in the overall CSP outcome or efficiency. More details about these analyses can be found in Figures S1 and S2.

Furthermore, assuming an explicit model of atomic polarizability in the force field CSP stage would lead to higher cost^52^ of that initial stage where we require low computational cost to enable thorough sampling of the crystal packing phase space. Previous investigations have shown that including the explicit polarizability does not have that significant effect on bulk phase energetics (unlike the inner dynamics^53^) as demonstrated by relatively similar performance of polarizable and nonpolarizable simulations of fusion enthalpies of ionic liquids.^52^ That would not justify the manifold cost increase of the initial generation stage, in particular in the case when subsequent QM refinement stages are to be included in the CSP funnel workflow. On the other hand, the average difference in fusion enthalpies between the polarizable and nonpolarizable models was found in at 3.7 kJ mol^–1^,^52^ which would interfere with the polymorph ranking in most cases of organic molecular crystals.

At a first glance, one can see that the PBE-based reoptimization places the experimental structure at the very top of the stability ranking. Also notable is the fact that the experimental structure, corresponding to the global energy minimum, is energetically separated from the rest of the landscape, with a considerable gap (about 2 kJ mol^–1^) between it and the next best structure in terms of *E*_el_. Additionally, we included a simple comparison of a generated XRPD pattern for this optimized structure versus the known experimental data,^6^ which can be accessed in the SI in Figure S13.

Although considering the static electronic energy only is not sufficient for a proper establishment of thermodynamic relationships among the crystal structures, this ranking serves as a good starting point for further computational methods. We took several structures (stemming from both [emIm] conformations) and calculated the thermodynamic contributions of their vibrational modes via the harmonic treatment as described in row two of Table 2 and in Section 2.2. The resulting harmonic vibrational Gibbs energy placed the experimental structure again at the best position within the ranking – the relative differences in these Gibbs energies in comparison with the other structures are presented in Figure 4 (right) at *T* = 200 K, as well as their evolution with temperature in Figure 5 (left). The considerable energy gap between the lowest energy structure and the other generated structures persists at 0 K, but decreases for certain structures to the point of becoming more stable than the lowest energy structure at temperatures close to 300 K. This steep decline of the Δ*G*(*T*) profiles is due to a higher entropy of the other predicted polymorph candidates when compared to the lowest-energy structure – an observation matching the available experimental data, where at least two high-entropy phases were observed – see phases crI and crIII in Figure 4 of ref 6. In the presented generated landscape, there are more candidates for these high-entropy phases and their “transition temperatures” are quite similar, which is why we decided to perform a more rigorous assessment of the thermodynamic contribution of the vibrational modes under the QHA.

**Figure 5:**
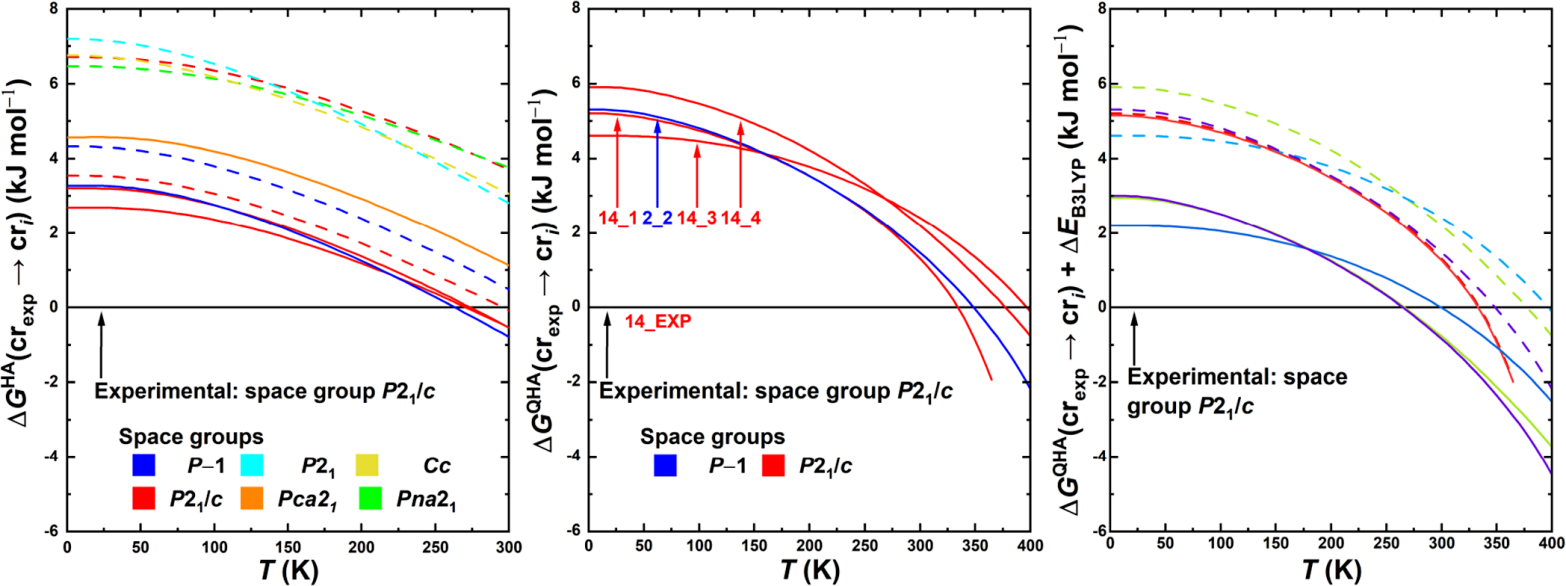
Results of the in silico polymorph ranking for [emIm]­[MeSO_3_] in terms of the Gibbs energy profiles of individual predicted crystal structures relative to the experimentally resolved stable structure as a function of temperature. Left box: results of the harmonic approximation. Middle box: results of the quasi-harmonic approximation for a narrowed set of structures (depicted in Figure 6). In the left and middle figures, full lines denote structures with [emIm] in Conformation 1 present, whereas dashed lines denote that Conformation 2 is present instead. Right box: results of the quasi-harmonic approximation including a B3LYP refinement of the electronic energies (solid lines) compared with PBE electronic energies (dashed). The color-coding denotes which pairs of lines correspond to the same structures of [emIm]­[MeSO_3_].

However, due to the fact that the QHA is much more computationally demanding than the simple harmonic approximation, we decided to narrow the list of the prospective structures for further QHA modeling one last time.

Computed *E*(*V*) curves are shown in Figure S3. Such functions mimic how the polymorph structures may look like at elevated temperatures or pressures. They also show that, even at slightly expanded volumes, the lowest energy structure [emIm]­[MeSO_3_] still wins over other generated structures. However, for the highly expanded unit cells, its *E*_el_(*V*) becomes comparable with certain structures, giving the possibility for a transition to take place between it and one of these structures, if this volume expansion were to be reached via an increase in temperature. This means only a handful of structures were selected to be treated at the level of theory in accordance with row four of Table 2, which is described in detail in Section 2.2. The results of this calculation are presented in Figure 5 (middle).

The QHA assessment did not change the fact that the experimental structure is placed at the best position within the energy ranking, particularly at 0 K. However, the QHA model shifts the predicted phase-transition temperatures from the low-temperature phase to the high-temperature phases toward higher temperatures, thus to a closer agreement with the experiment. Our QHA model currently predicts the lowest-lying solid–solid transition temperature from the experimentally resolved structure to a high-entropy monoclinic polymorph candidate structure at 335 K with another triclinic candidate structure predicted to take over the low-temperature form at 355 K. Comparison with the earlier experimental phase-behavior study of [emIm]­[MeSO_3_][Bibr ref6] reveals a reasonable qualitative agreement of our QHA model with experimental behavior of this IL. Experiments revealed that there were indeed two high-entropy phases taking over the low-temperature form in terms of their Gibbs energy in the temperature interval between 305 and 320 K (see Figure 7). Currently predicted phase transition temperatures are thus shifted by some 30 K to higher temperatures, which is, however, a very good computational achievement. Note that reaching a closer agreement in terms of these transition temperatures would require convergence of polymorph Gibbs energies well within the sub-kJ mol^–1^ accuracy region, which is extremely challenging to reach,^50^ requiring an even higher computational time investment than the present methodology. Still, based on this thermodynamic ranking, in particular the hierarchy of Gibbs energies, entropies and phase-transition temperatures, we can propose predicted structures 14_1 and 2_2 (see Figure 6) to match the calorimetrically detected forms crI and crIII, respectively. Due to the fact that the experimental high-entropy phases were only ever observed in a calorimeter and crystallized at too low temperatures, samples for X-ray measurements were not possible to be obtained in a sufficient quality and thus their experimental crystal structures are currently unknown.^6^ In this context, the structures in Figure 6 serve as the suggested theoretical structures for these unknown phases. Also note that there are additional predicted monoclinic candidate structures, being 0.9–1.3 kJ mol^–1^ higher in their Gibbs energies that the structure 2_2, which would correspond to phase-transition temperature higher by roughly roughly 25–40 K, illustrating the high sensitivity of the transition temperatures on the Gibbs free energy differences.

**Figure 6:**
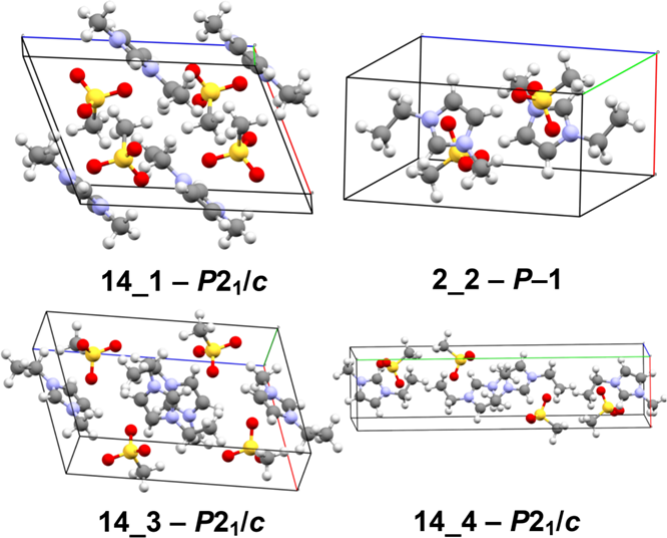
Four possible candidate polymorph structures identified among our predicted *Z*′ = 1 structures for [emIm]­[MeSO_3_] that could match the low-energy high-entropy experimentally observed polymorphs with yet unresolved crystal structures.

A closer inspection of Figure 7 reveals that the ambient-temperature enthalpy of the predicted candidate polymorphs 14_1 and 2_2 is overestimated by up to 1.5 kJ mol^–1^ with respect to the low-temperature form. At the same time, the entropy difference between those two high-entropy structures and the latter form are somewhat overestimated as indicated by steeper Δ*G*(*T*) profiles obtained from our QHA calculations when compared with the experimental Δ*G*(*T*) profiles taken from ref 6. This scenario leads to some fortuitous error cancellation between the enthalpic and entropic terms, improving the agreement of theory and experiment in terms of the phase-transition temperatures.

**Figure 7:**
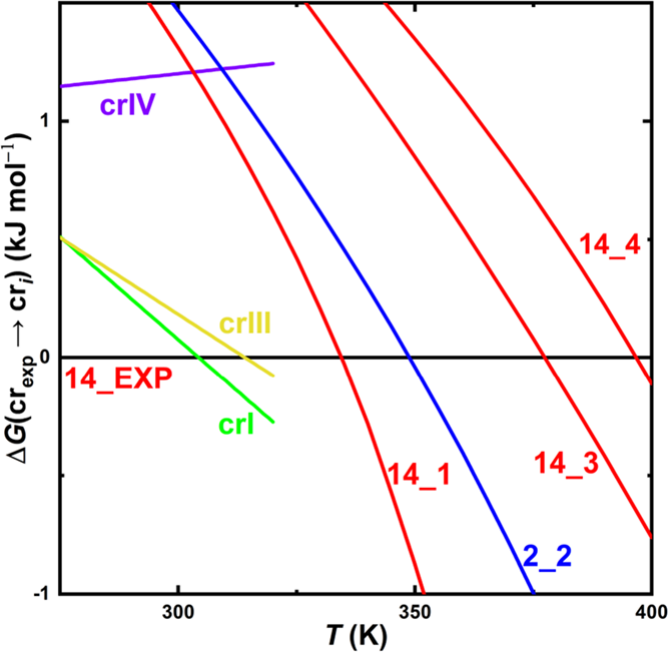
Comparison between the QHA-computed Gibbs free energies for selected predicted crystal structures of [emIm]­[MeSO_3_] and the experimental data (crI, crIII and crIV) gathered via DSC.^6^ Reference level of zero Gibbs energy change corresponds to the experimentally observed and resolved polymorph (labeled crII). Phases crI and crIII are reported to possess high entropy, whereas crIV is a low-entropy phase.

Comparison of the QHA results for the low-temperature polymorph with experimental data^6^ indicates a considerable computational underestimation of the heat capacity and entropy of the low-temperature form. Figure S12 illustrates a roughly 15% underestimation of the isobaric heat capacity. Indeed, the QHA coupled with such DFT levels of theory is known to systematically underestimate the isobaric heat capacity of various types of molecular crystals with errors ranging to 5–15%.^45,51,54^ Despite this considerable offset, one can presume that the QHA model consistently underestimates the heat capacities and entropies of all polymorphs to a comparable extent,^50,55^ again contributing to some error cancellation in construction of the Δ*G*(*T*) profiles.

On the other hand, predicted finite-temperature equilibrium densities of [emIm]­[MeSO_3_] (listed in Table S10) indicate that the found experimental structure exhibits the highest density, that being 1.342 g cm^–3^. This value is in a particularly good agreement with the experimental value of 1.370 g cm^–3^, being underestimated by about 2%, which corresponds to the typical QHA performance when coupled with the PBE-D3­(BJ)/PAW theory.^51,56^

There are several possible causes for this outcome from the computational point of view. Notably, the overestimation of vibrational frequencies can lead to the underestimation of the isobaric heat capacity. The density of states (DOS) functions of all the QHA-studied phases can be found in Figures S4 and S5, which do not seem to exhibit any obvious extremities. However, if any of the phonons involving vibrations of the heavy atoms (i.e., the S–O and S=O moieties of [MeSO_3_]) were severely overestimated, the impact on the resulting isobaric heat capacities would be significant. Moreover, the underestimation of all phonon frequencies seems to be systematic, as the heat capacities of the other generated phases coincidentally agree more with the known experimental data – meaning that it is highly likely that the whole set of functions is simply shifted downward. In essence, the qualitative assessment provided by this analysis is correct – structure “14_EXP” has the lowest heat capacity of all studied structures. That corresponds to the highest lattice mode frequencies, agreeing also with the strongest cohesive interactions among the [emIm] and [MeSO_3_] ions in that structure. And indeed, experimental data^6^ suggest that this phase should have the highest enthalpy of fusion of all the polymorphs, which is also reproduced in our data, as can be seen in Figure S12, where the “14_EXP” enthalpy curve is generally well below the others.

Lastly, we explored the effect of the simple B3LYP correction we postulated in Section 2.2 (last row of Table 2) on the ranking results. Figure 5 (right) displays the results of applying this correction. Due to its immense cost, the correction was only applied to the most promising structures of [emIm]­[MeSO_3_], i.e., the structures pictured on Figure 5 (middle). These curves are pictured again in Figure 5 (right) as dashed lines with a different color scheme and the B3LYP-corrected lines as solid lines. This time, the colors denote which pairs of lines computed on the two levels of theory correspond to the same structure.

The correction itself is not negligible with the exception of a single structure. In the other cases, it is larger than 2 kJ mol^–1^ and stabilizing the computed structures relative to the experimental structure. Though this means the stability of the experimental structure is no longer stable “by a large margin”, the fact that there is still a Gibbs energy gap larger than 2 kJ mol^–1^ at low temperatures still proves to be sufficient for the experimental structure to be more stable than any other discovered structure with *Z*′ = 1. Due to the performance of this correction for these structures, we decided to continue using it for other investigated cases as well.

Additionally, we also constructed a semiexperimental Gibbs energy profile for [emIm]­[MeSO_3_] liquid and mapped it onto our B3LYP polymorph ranking, which can be found in Figure S14. The liquid curve was constructed from the known experimental data,^6^ i.e., the enthalpy of fusion and the melting temperature of the most stable polymorph (crII) which enables to deduce its melting entropy, thus the slope of the liquid curve with respect to that of crII. The liquid curve was then constrained to intersect with the calculated Gibbs energy profile of the phase crII at its experimental melting temperature, i.e., at 309.5 K.^6^ Experimental data on the melting temperatures exhibit such a trend: *T*_m,crIII_ < *T*_m,crII_ < *T*_m,crI_. Notably, the current semiexperimental estimate of the melting temperature of the high-entropy crI form agrees on being the highest among the considered polymorphs, which further supports our claim that one of the two particular predicted structures would represent the crI form. In addition, the ordering mismatch of the semiexperimental melting temperatures of crII and crIII forms illustrates how challenging it is to quantitatively predict melting temperatures of polymorphs ordering. Note that an immense computational accuracy of the underlying Gibbs energies (tolerating uncertainties only in the sub-0.1 kJ mol^–1^ region that is currently inaccessible) would be needed to capture the sub-Kelvin differences of the melting temperatures of individual polymorphs.

### 3.2 Crystal Structure Prediction for [emIm]­[EtSO_4_]

Being satisfied with the performance of both the structure generation and the structure reranking methodology developed and tested for the case of [emIm]­[MeSO_3_], we deployed the same methodology for [emIm]­[EtSO_4_] as well. Even though the [EtSO_4_] is a slightly larger ion than the simpler [MeSO_3_] ion, still the ethyl moiety cannot be considered as a long largely flexible chain, so we treated it as rigid during crystal structure generation, as we stated in Section 2.1. Again, after generating 200 000 structures (100 000 for each [emIm] conformation), about 75 000 structures were found to be unique over the ten space groups. Comparing with the 35 000 thousand structures of [emIm]­[MeSO_3_], this follows chemical intuition that the larger molecule is simply going to have more original ways to create packing motifs, even given the space group restriction.

Combining these data with the FIT force field energy, we obtained the first idea of how the polymorph landscape can look for this compound, which can be found in Figure 8 (left). The overall shape of the landscape is similar in the high-energy region to what was observed for [emIm]­[MeSO_3_]. However, an important difference emerges in the low-energy region. Among the best-ranked structures, in the lowest 10 kJ mol^–1^ window, there are several structures with varying densities, yet similar energies. However, as was observed with [emIm]­[MeSO_3_], the force field energies are more-or-less a guideline in this context, and the following PBE reoptimization needs to be performed to verify the shape of the predicted landscape and the ranking of putative structures therein.

**Figure 8:**
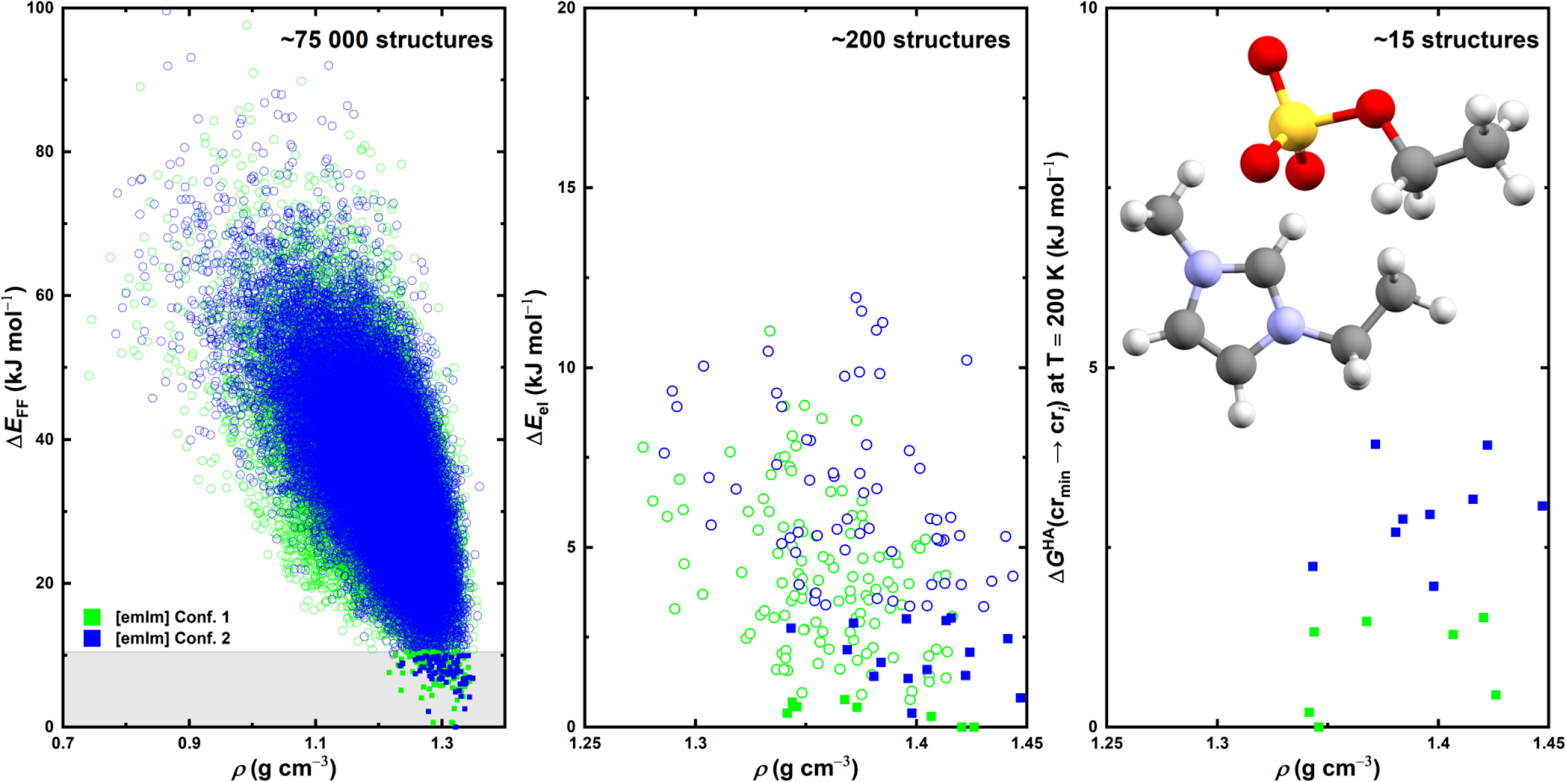
Polymorph landscapes predicted in the CSP process for [emIm]­[EtSO_4_] illustrating the shortlisting of predicted structures throughout the CSP funnel scheme. Left: The global [emIm]­[EtSO_4_] landscape generated using the mol-CSPy program with relative energies Δ*E*_FF_ corresponding to the FIT force field. The gray bar helps denote the energy of the last structure considered in further calculations. Middle: The landscape of the selected [emIm]­[MeSO_3_] structures from the figure on the left (full squares) when put through a PBE-D3­(BJ)/PAW reoptimization. Right: The Gibbs energy computed on the harmonic approximation level at *T* = 200 K for selected structures (full squares) from the figure in the middle. Hollow points in all boxes denote structures that were discarded from further modeling after the particular calculation steps, while solid squares denote structures that were selected for further analysis in subsequent steps.

Therefore, we chose the 10 kJ mol^–1^ window stemming from both [emIm] conformations to undergo reoptimization with PBE according to the methodology described in the first row of Table 2. The result of this reoptimization can be found in Figure 8 (middle). This time, there are even more structures with a similar energy and even larger differences in density than in the case of the force field ranking. For example, within the ≈2 kJ mol^–1^ gap between the lowest energy structure and the second lowest energy structure found in the [emIm]­[MeSO_3_], about ten structures can be found in the case of the [emIm]­[EtSO_4_] ranking. There are still multiple low density structures that achieved a low energy in the PBE ranking in comparison with the [emIm]­[MeSO_3_] landscape – this might suggest that there are some intermolecular interactions which facilitate crystals with more free space in between the molecules or that the size of the [EtSO_4_] ion has a massive influence on the crystal packing. Overall, the polymorph landscape can be at this stage described as “glassy” or “glass-like”, as the bulk phase has a lot of options to choose from during its hypothetical crystallization that appear to be energetically equally favorable.

This still holds when assuming the harmonic treatment of phonons, computed as described in the previous sections – graphs depicting the results of these calculations can be found in Figures 8 (right) and 9 (left). It shows that there is only weak to no temperature dependence of the ranking order, coming in stark contrast with the previous case, further suggesting that it is significantly more difficult to choose a stable polymorph from a multitude of alike structures. Additionally, the energy differences between the individual curves are much smaller compared to the previous case.

Continuing with our established methodology, we chose several structures to undergo the quasi-harmonic treatment to rigorously check our suspicions about the “glassy” quality of this landscape. The *E*(*V*) curves computed for the selected structures can be found in Figure S6. Again, the differences between the two sets of *E*(*V*) curves for both compounds is immediately noticeable – the *E*(*V*) curves for [emIm]­[EtSO_4_] predicted polymorphs are largely overlapping. Specifically, even the minima of the *E*(*V*) curves for one of the [emIm] conformations are found in close proximity of each other. Combining these curves with the vibrational analysis results in the free energy as a function of temperature, which can be found in Figure 9 (middle). These results lead to the same conclusion: the landscape appears glassy and there is virtually no universally stable structure that would stand out against the others.

**Figure 9:**
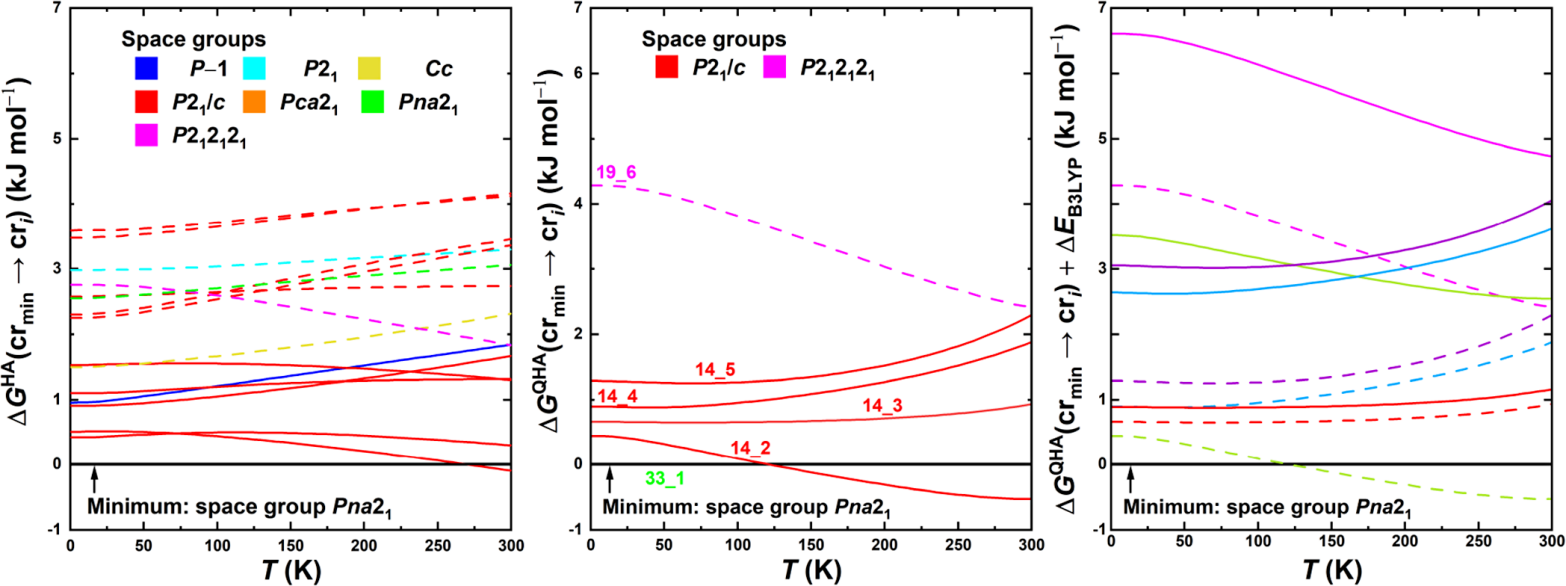
Results of the in silico polymorph ranking for [emIm]­[EtSO_4_] at finite temperatures. Left: Gibbs energy profiles of individual prospect crystal structures relative to the experimentally resolved stable structure as a function of temperature computed via the harmonic approximation. Middle: The same function, computed instead via the quasi-harmonic approximation and for a narrowed set of structures. Right: Comparison of the Gibbs energy profiles of selected candidate polymorph structures of [emIm]­[EtSO_4_] relative to the experimentally resolved stable structure computed using PBE (dashed lines) and the B3LYP correction (solid lines). The color-coding denotes which pairs of lines correspond to the same structures of [emIm]­[EtSO_4_]. In the left and middle figures, full lines denote structures with [emIm] in Conformation 1 present, whereas dashed lines denote that Conformation 2 conformation is present instead.

As was the case in the previous section, we decided to employ the B3LYP correction to structures of [emIm]­[EtSO_4_] as well. Figure 9 (right) contains the results of this comparison. Note that the same modifications to line styles and line colors apply as in the previous section. Recall that in the case of [emIm]­[MeSO_3_], the correction *stabilized* the structures relative to the experimental structure. In this case, the majority of the corrections are larger than approximately 1.5 kJ mol^–1^ and are *destabilizing* the structures relative to the minimum energy structure. This slightly changes the outlook on the landscape, but the point still stands that even according to the B3LYP correction, one of the structures is only less than 1 kJ mol^–1^ above the minimum structure and thanks to the previously computed quasi-harmonic evaluation, we know that there is no stark entropy-related stabilization at higher temperatures. Therefore, the notion that the polymorph landscape still retains its glassy shape after assuming all relevant corrections possibly explains the reluctance of this IL to crystallize. Unit cells of six most promising predicted [emIm]­[EtSO_4_] structures with *Z*′ = 1 are depicted in Figure 10.

**Figure 10:**
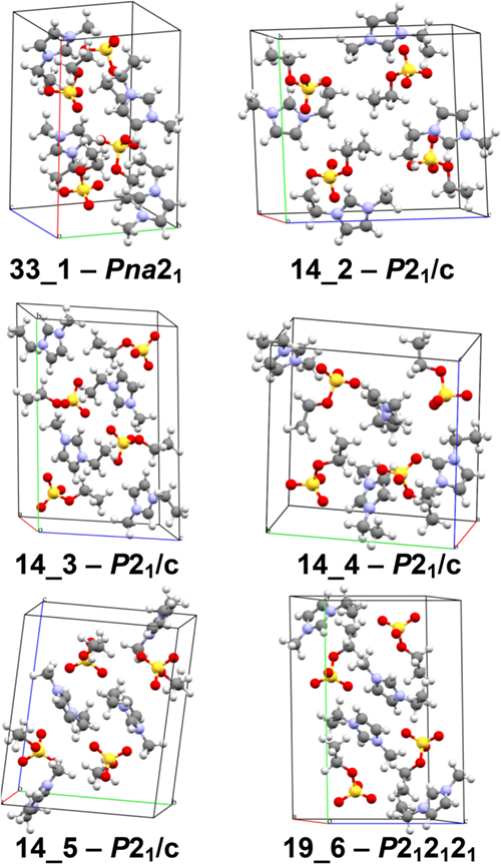
Selected highlighted [emIm]­[EtSO_4_] structures from Figure 9 (middle).

### 3.3 Crystal Structure Prediction for *Z*′ > 1 Crystals of Ionic Liquids

In order to increase our possible search space and therefore ensure that the potential energy landscape was well-searched, we decided to explore possible *Z*′ > 1 crystal structures of both systems and specifically, we decided to focus on *Z*′ = 2 structures. According to our searches in the CSD,^29^ there are several *Z*′ > 1 structures of [emIm] that contain at the same time *different* [emIm] conformations within their lattice, as well *Z*′ > 1 structures that simply contain multiple independent copies of the same conformation. Some examples include: REFCODEs VEPFOL02 and VEPFOL03 (both *Z*′ = 4), DOJNUM, ILELAO, KUCPED01 through KUCPED11, and RENSEJ01 (all *Z*′ = 2), which feature the ethyl group pointing both away or toward the methyl group on the other side of the ring, though it also points further away from the ring plane, likely caused by either the small size of the anion, presence of aromatic rings on the anion or other intermolecular interactions. Since the molecules are treated as rigid within any one run of the structure generation algorithm, we elected to run three additional *Z*′ = 2 searches for each of the compounds: two where only one of the [emIm] conformations was considered and one run which considered both. This makes six additional runs in total.

Moreover, we also modified the search criteria in terms of the space groups which were considered in the search, so that we again captured the most prominent *Z*′ > 1 space groups within the CSD. These space groups were *P*1 (1), *P*–1 (2), *P*2_1_ (4), *P*2_1_/*c* (14), and *P*2_1_2_1_2_1_ (19).^29^ As before, we generated ten thousand energy minimized crystal structures in each of these space groups.

Figure S9 shows force-field-based polymorph landscapes for both compounds with the *Z*′ = 2 structures included. As can be seen, some higher *Z*′ structures do indeed get close to the best *Z*′ = 1 structures predicted by the force field. Therefore, we decided for some of them to undergo reoptimization via PBE, specifically the lower 10 kJ mol^–1^ window from each of the six sets of structures, similar to the methodology we employed for the *Z*′ = 1 structures. Naturally, we did not perform the reoptimization for the structures from these windows that would symmetrically map onto other structures already generated and reoptimized within the *Z*′ = 1 set. However, since the *Z*′ = 2 structures are generally larger and thus present more demanding computations, we did not subject these structures to the full set of reoptimizations described in Section 2.2. Namely, we did not perform the calculations pertaining to the quasi-harmonic approximation and the B3LYP correction, i.e., the rows from three to five of Table 2.

Global results of the reoptimization are presented in Figure 11. Again, the differences between the two landscapes are quite noticeable – with the addition of the *Z*′ = 2 structures, the landscape of [emIm]­[EtSO_4_] became even more cluttered, with a lot of structures being now placed around the similar lowest energy with a large diversity of densities. The [emIm]­[MeSO_3_] results, on the other hand, place the *Z*′ = 2 structures at a similar energy above the lowest energy *Z*′ = 1 structure, showing that it is increasingly difficult to find a new area where to look for structures that would hypothetically be more stable than the experimentally observed stable structure.

**Figure 11:**
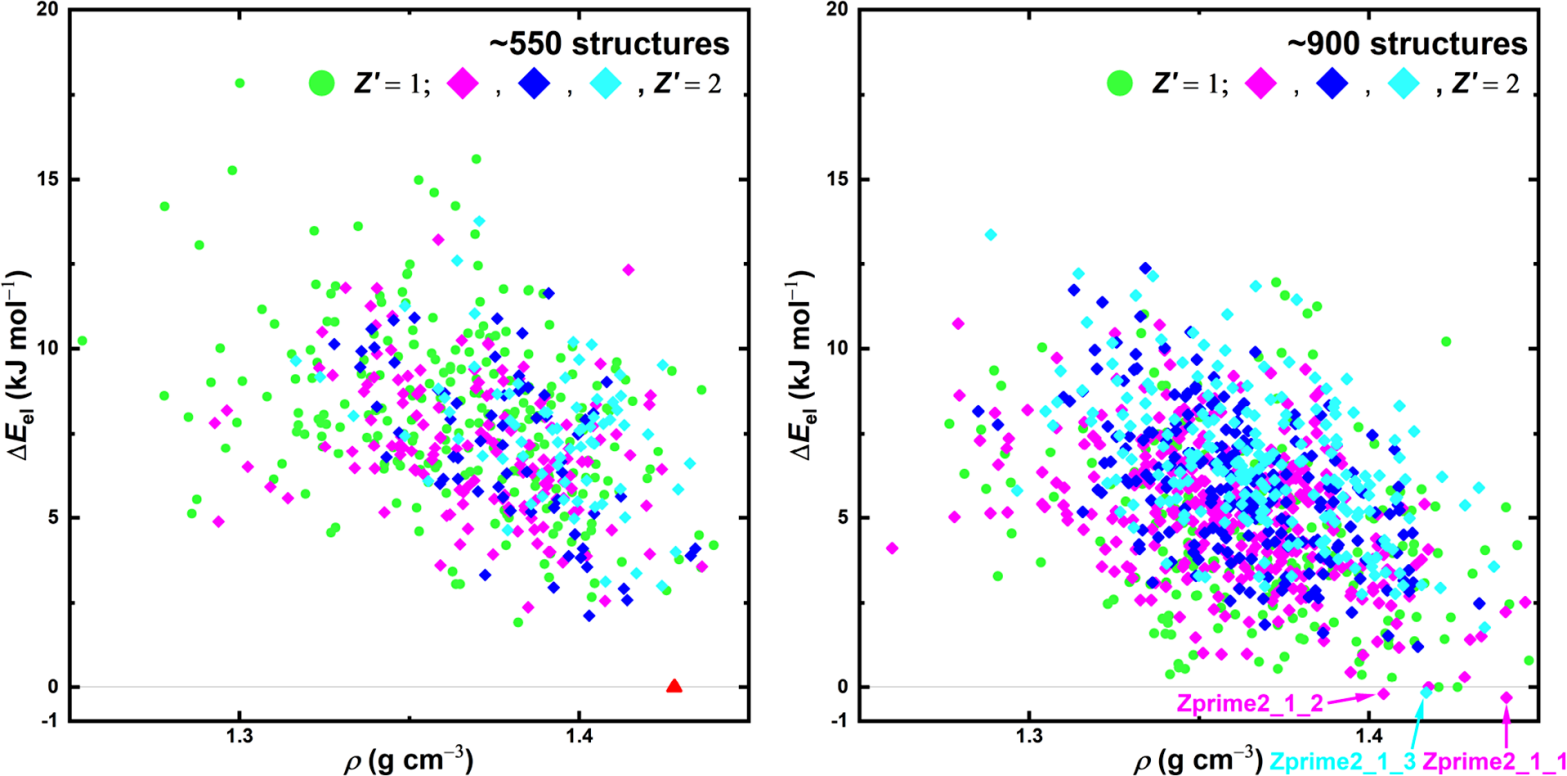
Basic PBE reranking results for the best [emIm]­[MeSO_3_] (left) and [emIm]­[EtSO_4_] (right), comparing *Z*′ = 1 and *Z*′ = 2 results. In the left figure, the red triangle denotes the experimental structure. The color coding for the *Z*′ = 2 structures represents what molecular conformations of [emIm] were used as input for generation: magenta – both from experimental crystal, cyan – both with rotation within the ethyl group, blue – mixed.

Nonetheless, we decided to also perform the simpler harmonic approximation analysis for some structures of [emIm]­[MeSO_3_], so that we could gauge the effect of phonons on larger crystal structures. We still followed the general guidelines we set out in Section 2.2 for the HA, except we applied it only to *Z*′ = 2 structures that were within 3 kJ mol^–1^ from the experimental structure. Accounting for structure duplicates with the *Z*′ = 1 set, this came out to only four structures, stemming from the calculation where two unique [emIm] conformations are present, which cannot be symmetrically equivalent to any *Z*′ = 1 structure.

Figure S10 presents the result of these calculations – the harmonic correction situated the *Z*′ = 2 structures even higher in Gibbs energy than the *Z*′ = 1 structures that featured the less favorable ethylmethylimidazolium conformation. Our above stated experience with QHA for the *Z*′ = 1 structures is such that QHA does not impart any important qualitative changes to the shape of the polymorph landscape and ranking with respect to the Gibbs energies established by the harmonic approximation (see Figures 5 and 9). More precisely, as can be seen in Tables S6 and S7, both at *T* = 0 K and *T* = 200 K, the Δ*G*(cr_a_ → cr_b_) actually increases, so it would not have the effect needed for the *Z*′ = 2 curves pictured in Figure S10 to become more stable than the found experimental structure.

For [emIm]­[EtSO_4_] structures generated to accommodate *Z*′ = 2, considering how little the rankings resulting from both the harmonic and quasi-harmonic approximations differed from the basic PBE calculations for the *Z*′ = 1 structures and how it performed for the [emIm]­[MeSO_3_] structures with *Z*′ = 2 in terms of the spread of the resulting differences, we did not perform any additional QHA calculations. The comparisons of these values can be accessed in Tables S8 and S9.

Instead, we postulate that the contributions from (quasi-)­harmonic vibrational modes for the *Z*′ = 2 structures are not expected to differ significantly from what was observed for the *Z*′ = 1 structures. Therefore, the polymorh ranking is likely to remain qualitatively similar even if full QHA treatment would have been performed for predicted *Z*′ = 2 structures of [emIm]­[EtSO_4_]. It is thus highly unlikely that any of the generated *Z*′ = 2 structures of [emIm]­[EtSO_4_] structures would stand out significantly in terms of the Gibbs free energy. Also, although there are numerous structures very close in terms of their energy with *Z*′ ≤ 2, it is practically impossible to precisely rank their stabilities only relying on a GGA-tier DFT functional without investing additional immense computational resources into this task. Unit cells of three most promising predicted [emIm]­[EtSO_4_] structures with *Z*′ = 2 are depicted in Figure 12.

**Figure 12:**
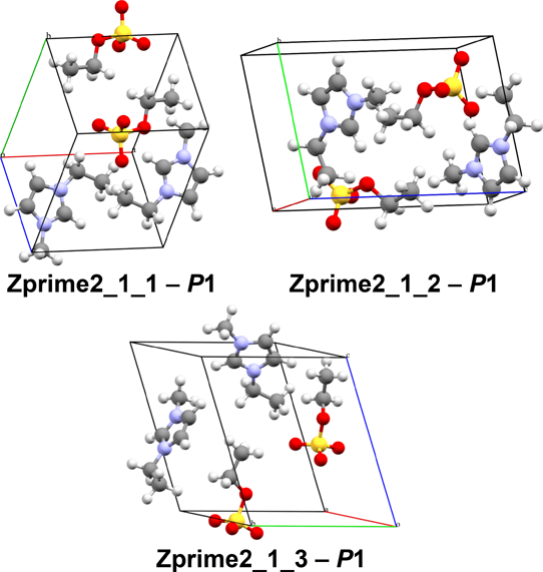
Selected [emIm]­[EtSO_4_] structures highlighted in Figure 11.

Overall, the addition of the *Z*′ = 2 structures did not qualitatively change the conclusions reached for either IL we studied, apart from providing higher confidence that the CSP study represents a near-complete study of the crystal structure landscapes of both ILs. For [emIm]­[MeSO_3_], the experimental structure which was also found within the course of the search is still the most stable out of all the ones we generated/analyzed with (Q)­HA. On the other hand, for [emIm]­[EtSO_4_], although we do observe several *Z*′ = 2 structures falling below the minimum energy structure(s) reached via the static PBE for *Z*′ = 1, the overall shape of the landscape still hints at the compound forming glassy rather than crystal phases.

Since there are organic materials with numerous known crystalline polymorphs of similar energies densely filling the lower-energy regions of the polymorph landscape,^57–59^ there has to be a particular phenomenon at play in this case which imparts a different behavior of the barely crystallizing ILs with predicted rich glassy polymorph landscapes. It has been shown that the existence of a glassy polymorph landscape can lead to pronounced disorder of organic molecular crystals.^60^ Considering the strong electrostatic cohesive interactions among explicitly charged molecules that constitute the bulk of ILs,^51^ one can assume that perturbation of a crystal structure required for a polymorphic transition would require a significant amount of energy, likely exceeding that relevant for similar rearrangements in crystals of neutral molecules. Therefore, we hypothesize that crystallization of [emIm]­[EtSO_4_] is impeded by too large energy barriers separating particular crystallite nuclei emerging rather randomly throughout the crystallization from the melt. Those nuclei then cannot unify their structure to grow a particular crystal structure as such rearrangements would need to overcome massive energy barriers.

Computational methods to model the energy barrier between distinct polymorphs do exist^61^ and have been used to rationalize the experimental crystallization conditions required to form particular polymorphs of simpler organic compounds.^62^ However, in the current case of [emIm]­[EtSO_4_], where DFT lattice energies are required, using such methods would currently be too costly and would far exceed the scope of the current study. Still, even without the explicit assessment of barrier heights, we believe that our results could be used to gain insights a priori. For example, the structures we highlighted as most stable for [emIm]­[EtSO_4_], while sharing similar energies, are structurally different. This is evidenced by a COMPACK similarity analysis^31^ that revealed massive structural dissimilarities among the most promising candidate structures predicted for [emIm]­[EtSO_4_] with no more than two molecules from the crystal structure being typically found as a structural match between low energy predicted structures. We believe that, had the individual structures been at least somewhat similar to each other, the resulting barriers among them might have been relatively small. However, the opposite is true: the large structural differences, combined with strong electrostatic interactions, supports our hypothesis of high energy barriers between these nearly equi-energetic structures.

## 4 Conclusions

We have presented a methodology for crystal structure prediction applicable for aprotic ionic liquids, both in terms of structure generation and polymorph stability reranking. Furthermore, we demonstrated that for a crystallizing ionic liquid, [emIm]­[MeSO_3_], the CSP methodology is able to find the only experimentally resolved crystal structure and the subsequent DFT-based QHA reranking puts it correctly as the global energy minimum, at the top of structural stability at low temperatures. Using thermodynamic arguments based on our QHA calculations of Gibbs energies and phase-transition temperatures of individual predicted structures, we also postulated candidate crystal structures which could correspond to other known high-entropy crystalline phases of [emIm]­[MeSO_3_]. Additional crystallographic experiments would be required to prove whether our proposed crystal structures match those high-temperature polymorphs in reality. Still, the described success of our CSP methodology with this compound is a step toward a crystal structure prediction methodology usable for various other materials.

Using the fact that the methodology successfully reproduced the most important part of the [emIm]­[MeSO_3_] phase behavior, we applied it to predict crystal structures of [emIm]­[EtSO_4_], which has no known experimental crystal structure as of yet. In the results of both the generation and reranking stages, we discovered that the polymorph landscape of [emIm]­[EtSO_4_] contains a multitude of structures that are very similar in their Gibbs energies. Since there is no structure that particularly stands out in terms of the relative stability, the generated polymorph landscape can be described as glassy. Crystal energy landscapes with many nearly equi-energetic local minima are not uncommon and, for neutral organic molecules, low energy barriers can allow a system to explore these minima during crystallization.^57,58^ For ILs with much stronger electrostatic interactions,^51^ these individual candidate polymorphic structures are expected to be separated by relatively high energy barriers due to strong local cohesive interactions in the ionic materials. We thus speculate that during a hypothetical crystallization, [emIm]­[EtSO_4_] may select from multiple nearly equi-energetic structures for nucleation as far as packing is concerned. Upon nucleation of such a solid ionic system from the melt, once individual ions attain some fixed, yet due to the equi-energetics rather arbitrary, local arrangement within the growing solid, the high energy barriers hinder their rearrangement to match any single target crystal structure. This would effectively impede the crystallization from the melt, locking the ions in an amorphous state, ultimately resulting in the vitrification of this IL upon cooling. These CSP conclusions on [emIm]­[EtSO_4_] agree with the yet unobserved crystallization of this compound at the time of writing. The precise nature of crystallization also depends on kinetic factors and it may be enabled in the future by proper seeding techniques or by crystallizations from other media than plain melt. Due to the glassy shape of the predicted polymorph landscape with multiple energetically comparable structures, also [emIm]­[EtSO_4_] can be expected to be highly polymorphic once one manages to prepare any crystalline form of that compound.

## Supplementary Material





## Notes

Detailed results on the electronic energy variation of selected predicted structures, their densities of phonon states, and optimized lattice parameters for the most promising structures (PDF)
Archive of the DFT-optimized predicted crystal structures for both target compounds (ZIP)
