# Supplementary material for: Crystal Structure Prediction for Aprotic Ionic Liquids – Searching for the Unknown
Source: Cryst Growth Des. 2026 Jan 19;26(3):1445–58. doi: 10.1021/acs.cgd.5c01674 (PMC12879539; doi:10.1021/acs.cgd.5c01674)
Supplement: Supplementary file 1 [file cg5c01674_si_001.pdf]

Crystal structure prediction for aprotic ionic liquids – searching for the unknown

Petr Touš, Graeme M. Day, Ctirad Červinka

Supplementary information

Contents

S1 Structure generation **3**

S2 Components and selected results of the QHA for selected [emIm][MeSO$_3$] crystals **5**

S3 Components of the quasi-harmonic approximation for selected [emIm][EtSO$_4$] crystals **8**

S4 Selected $Z' = 2$ results for both studied ILs **11**

S5 Lattice parameters for selected strutures of both studied ionic liquids **13**

S6 Comparison of various approximations to the Gibbs free energy **14**

S7 Comparison with known experimental data for [emIm][MeSO$_3$] **15**

S1 Structure generation

Table S1: Relative energies (in kJ mol^{-1}) for the two assumed conformations of [emIm] in the gaseous phase computed on two levels of theory. Note that the CCSD(T) energy was computed for a molecule optimized on the CCSD level of theory. All these calculations were made in the Gaussian program.[1]

Conformation	PBE/6-311+G(d,p)	CCSD(T)/aug-cc-pvdz
Conformation 1	0.0	0.0
Conformation 2	2.707	2.702

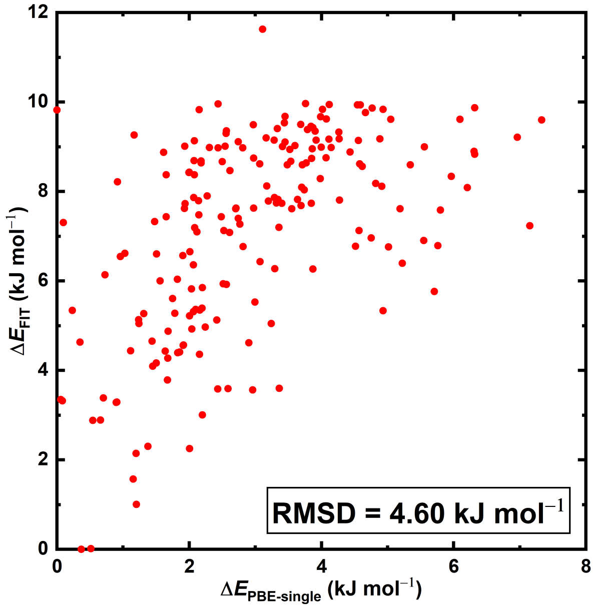

Figure S1: Correlation of relative energies for a subset of predicted [emIm][$MeSO_3$] crystal structures optimized using the FIT force field and evaluating the total energy based either on the FIT model or using PBE-D3 single-point calculations for the same structures (*without* optimizing the atomic coordinates or cell parameters, hence being denoted as "PBE-single").

Figure S1 shows a comparison of the relative energies of predicted [emIm][$MeSO_3$] crystal structures optimized at the FIT level of theory with PBE-D3 single-point energies evaluated for the same structures (ie. at the force field optimized geometries). Thanks to filtering out the impact of PBE-D3 structural optimizations, such an analysis illustrates the quality of the force field calculations (e. g. illustrating the lack of explicit polarizability effects) for the target compound benchmarking the FIT results against a more robust (yet still imperfect) quantum-chemical alternative. Figure S1 depicts data points for about two hundred different structures, with a mixture of $Z' = 1$ and $Z' = 2$ values – within the lowest 10 kJ mol^{-1} energy window as measured by PBE-D3 theory. The correlation between FIT and PBE-D3 energies is rather weak, however, that is not to be unexpected – for systems studied here, with significant molecular flexibility, net molecular charges and atom types that are not included in the original force field parametrization, the FIT force field is designed to be an inexpensive qualitative method to filter out obviously incorrect structures and provide reasonable starting structures for further refinement. Importantly, scatter of the data points on the relative polymorph energies from FIT and PBE-D3 theories considered in Figure S1 correspond to RMSD of 4.6 kJ mol^{-1} in the energetic ranking of both models. Due to these appreciable energetic shifts of individual predicted structures throughout the CSP funnel (reaching up to 9 kJ mol^{-1} for [emIm][$MeSO_3$]), one has to consider a significantly broader energy window with higher numbers of structures to be retained for each CSP funnel stage to ensure robustness of the CSP methodology and not to lose any viable crystal structures as the CSP funnel progresses. In this case, we retained

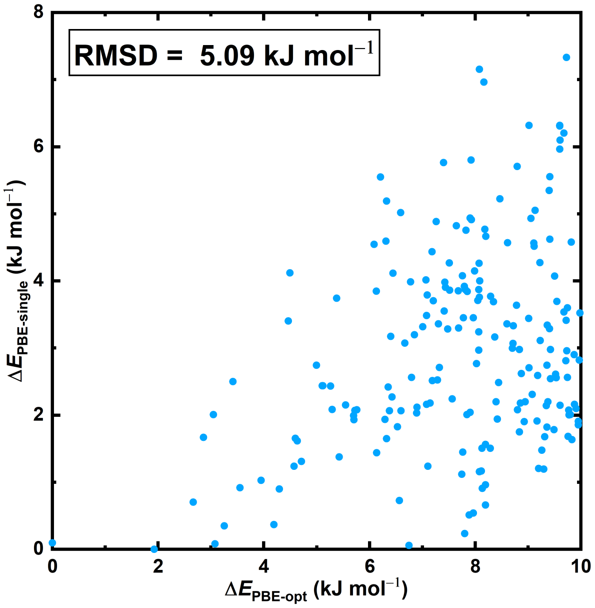

Figure S2: Correlation of relative energies for the same subset of structures as in Figure S1. This time comparing the single point PBE-D3 energies ("PBE-single") and the corresponding energies given by PBE-D3 after optimization.

over 500 structures for ranking on the PBE-D3 level of theory spanning up to a maximum ΔE of about 22 kJ mol^{-1} in the PBE energies so that these inconsistencies between FIT and PBE-D3 energies are expected to play a marginal role on the final results of the CSP process.

Similarly, we attempted to quantify the compound energetic effect of the difference in crystal geometries between the FIT generated structures and the PBE-optimized geometries. In Figure S2, we compare the single point PBE-D3 energies (corresponding to FIT geometries) and PBE-D3 energies following geometry optimization. Again, significant reordering can be observed upon optimization and the correlation of these data is quite weak, with RMSD being equal to about 5.1 kJ mol^{-1}. This means that the force-field-based generation produced structures that required noticeable relaxation. However, this is again an expected outcome; the force field is expected to identify basins on the polymorph landscape in order for the consequent relaxation to identify their minima. Given the fact the experimental structure (the solitary point on the far left of S2) was identified by this process, we again consider the 500 structures optimized on the PBE level of theory to be sufficient in identifying all important best-performers on the landscape.

S2 Components and selected results of the QHA for selected [emIm][$MeSO_3$] crystals

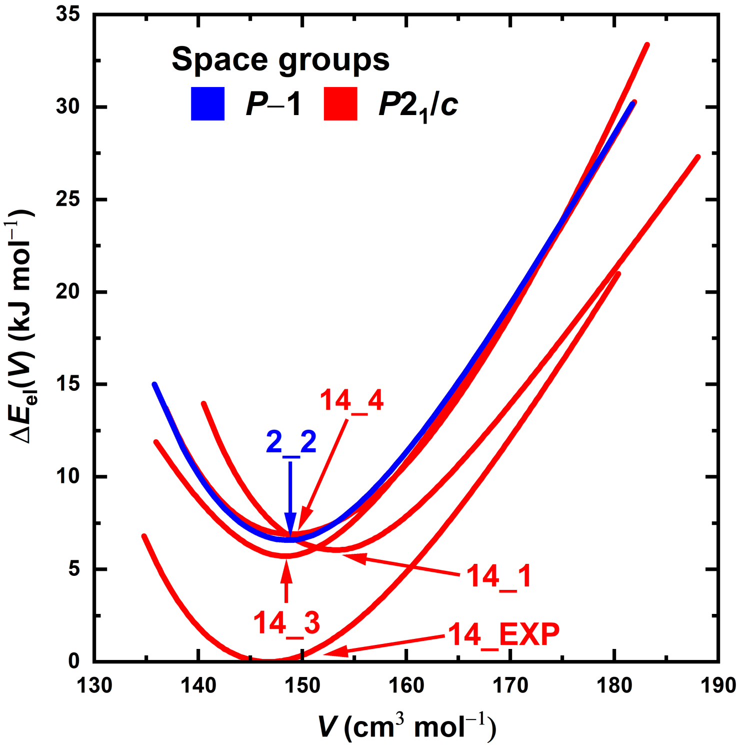

Figure S3: The $E(V)$ curves computed for selected structures of [emIm][$MeSO_3$] and fitted with the Murnaghan equation of state – the experimental structure corresponds to the lowest-lying curve. All of the curves pictured correspond to structures which underwent the next step in the quasi-harmonic approximation – the phonon analysis. All structures represented by the curves contain the axial [emIm] molecular conformation. The numbers assigned to curve minima denote structures to their corresponding numbers in Figures in the main text.

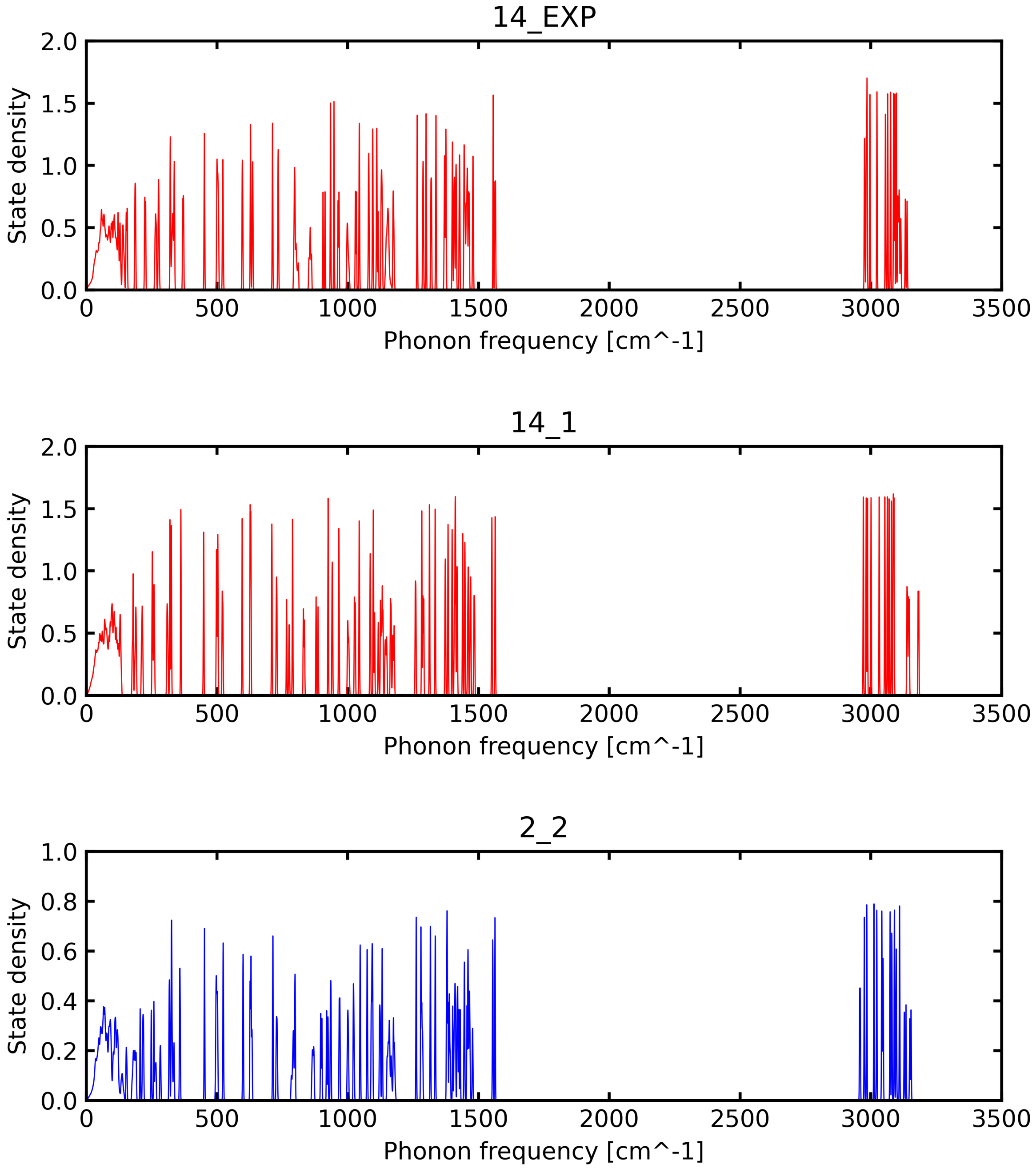

Figure S4: Density of phonon states for [emIm][$MeSO_3$] structures corresponding to minima on the $E(V)$ curve as pictured in Figure S3 and Figures in the main text. The notation refers to the space group and then the number assigned in Figure S3.

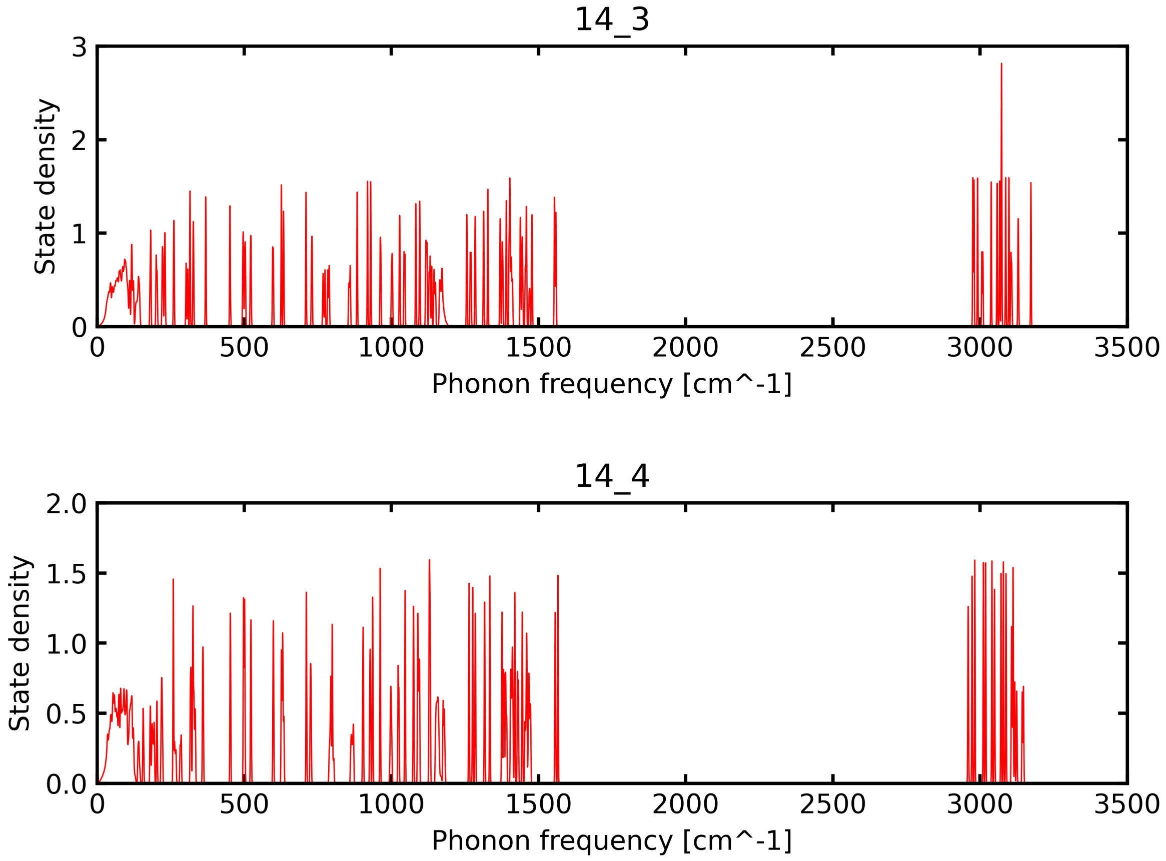

Figure S5: Continuation of Figure S2. Density of phonon states for [emIm][$MeSO_3$] structures corresponding to minima on the $E(V)$ curve as pictured in Figure S3 and Figures in the main text. The notation refers to the space group and then the number assigned in Figure S3.

S3 Components of the quasi-harmonic approximation for selected [emIm][EtSO$_4$] crystals

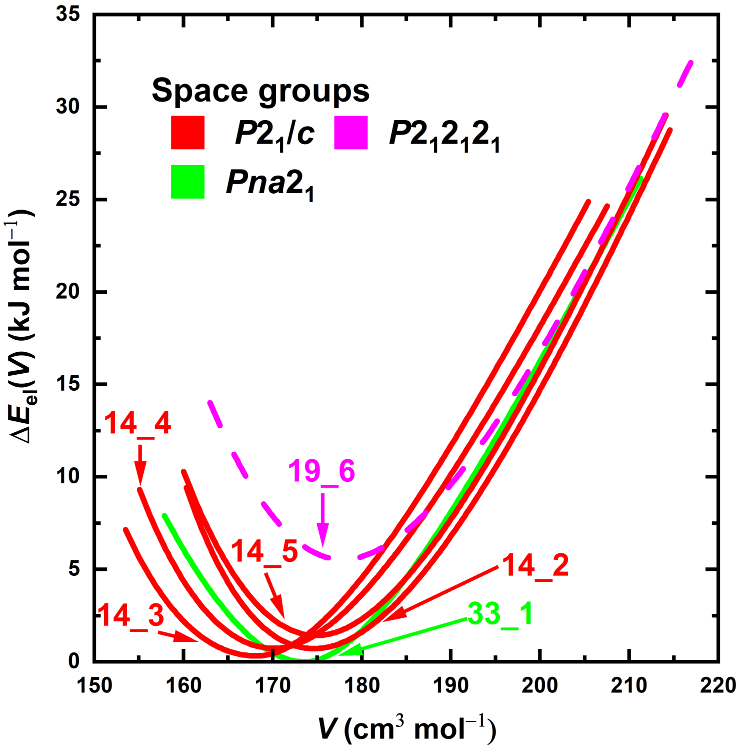

Figure S6: The $E(V)$ curves computed for selected structures of [emIm][EtSO$_4$] and fitted with the Murnaghan equation of state. All of the curves pictured correspond to structures which underwent the next step in the quasi-harmonic approximation – the phonon analysis. All structures represented by the curves with the exception of one (dashed line) contain the axial [emIm] molecular conformation.

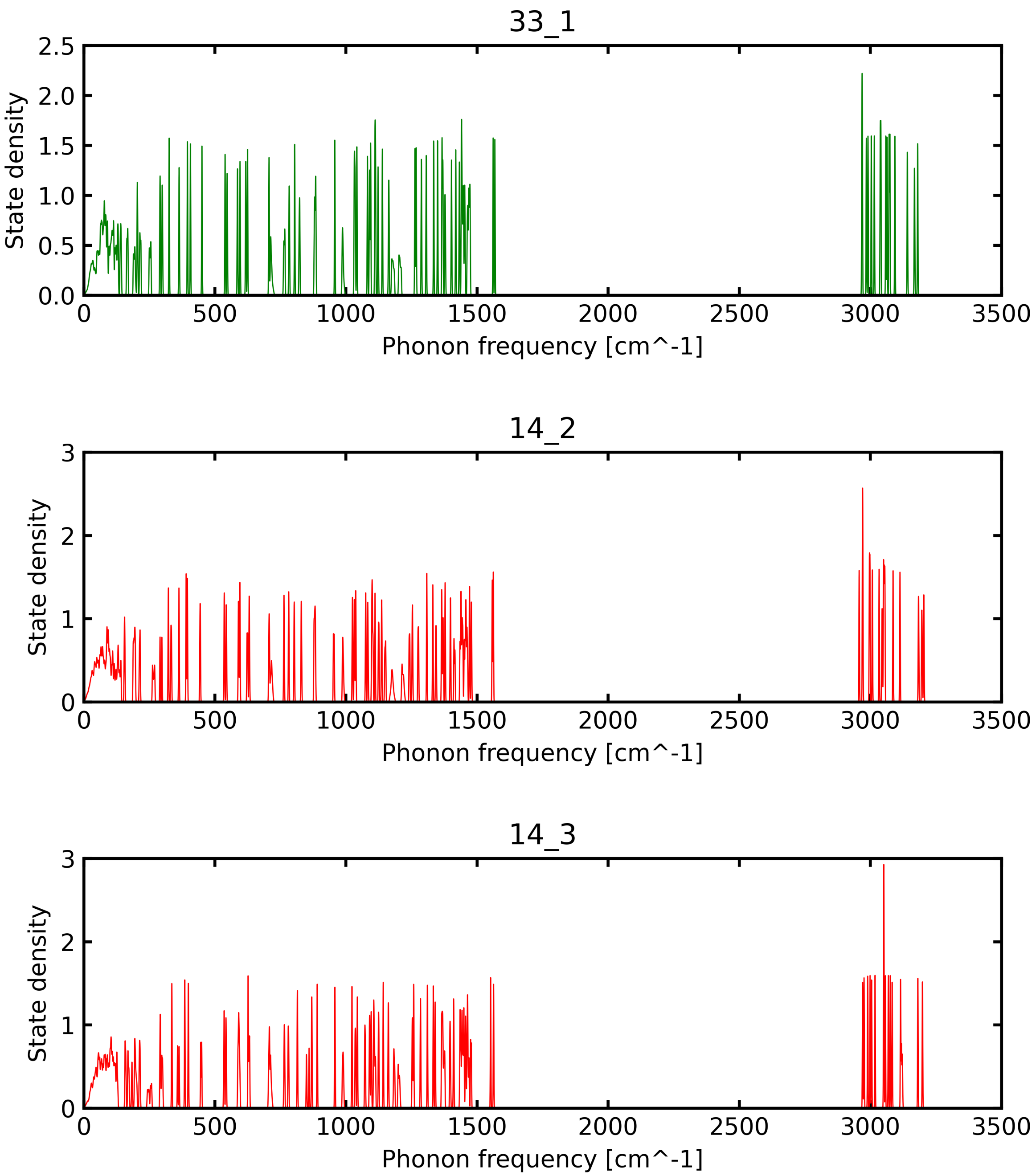

Figure S7: Density of phonon states for [emIm][$EtSO_4$] structures corresponding to minima on the $E(V)$ curve as pictured in Figure S6 and Figures in the main text. The notation refers to the space group and then the number assigned in Figure S6.

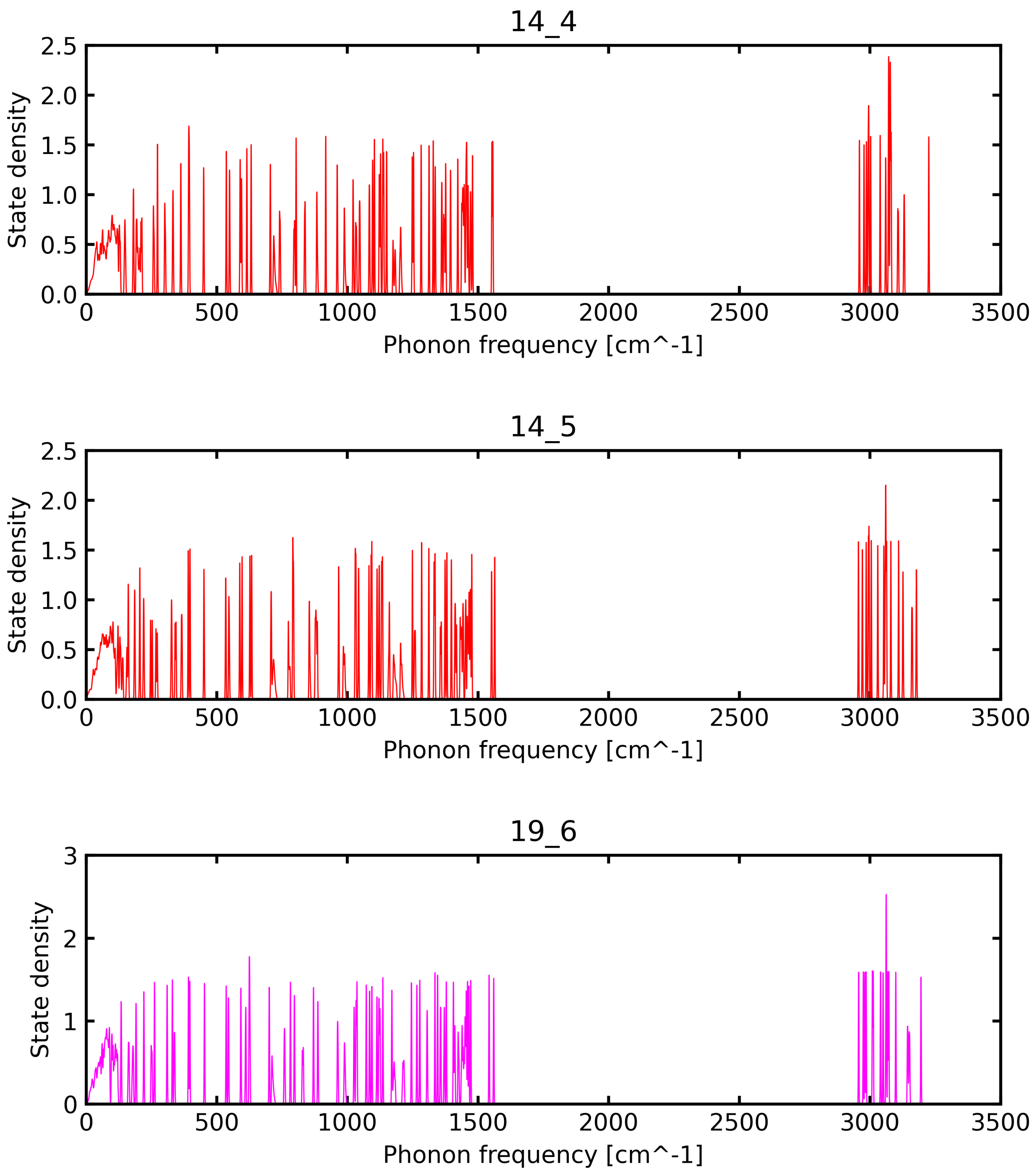

Figure S8: Continuation of Figure S5. Density of phonon states for [emIm][EtSO$_4$] structures corresponding to minima on the $E(V)$ curve as pictured in Figure S6 and Figures in the main text. The notation refers to the space group and then the number assigned in Figure S6.

S4 Selected $Z' = 2$ results for both studied ILs

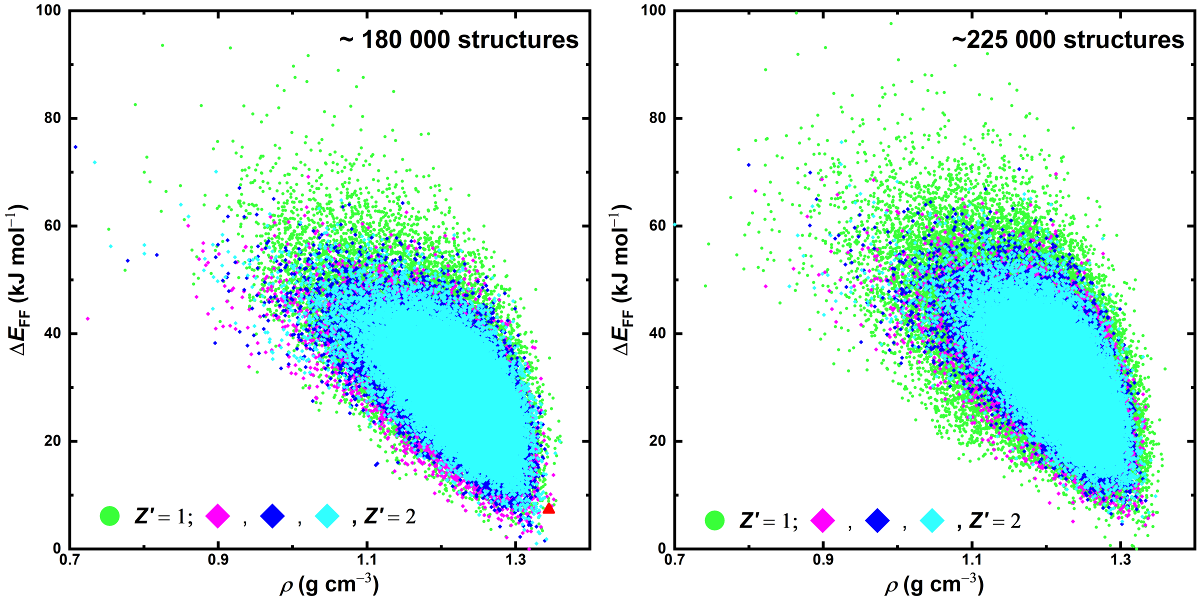

Figure S9: Force-field-based rankings for [emIm][MeSO3] (left) and [emIm][EtSO4] (right), comparing $Z' = 1$ and $Z' = 2$ results. In the left figure, the red triangle denotes the experimental structure. The color coding for the $Z' = 2$ structures represents what molecular conformations of [emIm] were used as input for generation: magenta – both axial, cyan – both equatorial, blue – mixed.

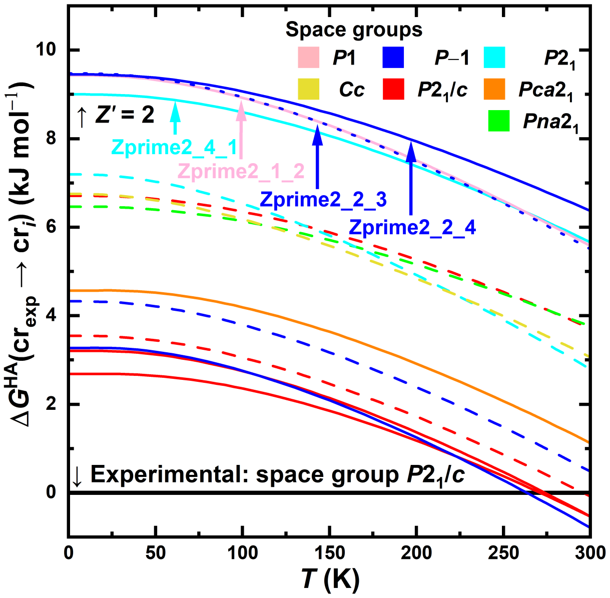

Figure S10: The Gibbs energy ranking computed on the HA level for [emIm][$MeSO_3$] including several $Z' = 2$ structures. The featured $Z' = 2$ structures include both assumed [emIm] conformations within its crystallographic unit.

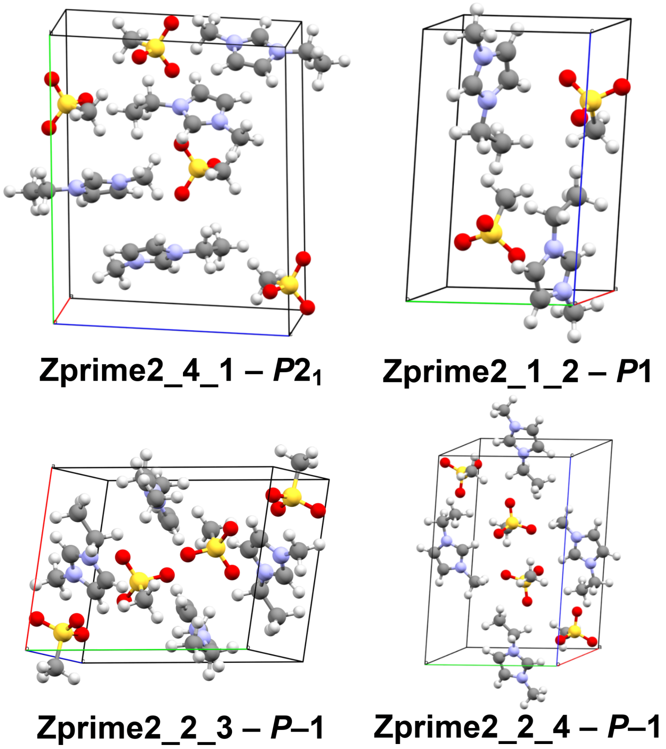

Figure S11: Crystal structures for selected $Z' = 2$ structures of [emIm][$MeSO_3$] highlighted in Figure S10.

S5 Lattice parameters for selected strutures of both studied ionic liquids

Please note that for all the Tables in this section, the lattice parameters are given either in Å (for a, b and c) or in degrees (for α, β and γ).

Table S2: Lattice parameters for [emIm][$MeSO_3$] structures highlighted in Figure S3.

Name	a	b	c	α	β	γ
SAGPOJ[2]	5.583	12.020	14.978	90.00	90.00	95.75
14_EXP	5.579	14.720	12.005	90.000	96.313	90.000
14_1	7.660	12.972	11.216	90.000	113.550	90.000
2_2	5.846	7.157	12.034	82.438	89.256	81.521
14_3	7.994	9.219	14.078	90.000	107.715	90.000
14_4	5.777	24.069	7.240	90.000	98.776	90.000

Table S3: Lattice parameters for [emIm][$EtSO_4$] structures highlighted in Figure S6.

Name	a	b	c	α	β	γ
33_1	14.399	9.136	8.725	90.000	90.000	90.000
14_2	7.062	12.759	14.009	90.000	112.567	90.000
14_3	6.748	16.921	11.674	90.000	123.145	90.000
14_4	7.905	16.581	10.534	90.000	125.259	90.000
14_5	7.469	11.561	14.512	90.000	111.813	90.000
19_6	6.914	13.001	13.182	90.000	90.000	90.000

Table S4: Lattice parameters for $Z' = 2$ [emIm][$MeSO_3$] structures highlighted in S10.

Name	a	b	c	α	β	γ
Zprime2_4_1	5.747	14.506	12.042	90.000	98.302	90.000
Zprime2_1_2	5.776	7.226	12.095	94.366	97.846	99.074
Zprime2_2_3	9.258	11.075	11.434	61.831	76.325	78.269
Zprime2_2_4	6.759	9.708	15.621	95.790	90.603	105.398

Table S5: Lattice parameters for $Z' = 2$ [emIm][$EtSO_4$] structures highlighted in the main text.

Name	a	b	c	α	β	γ
Zprime2_1_1	7.164	9.019	9.065	81.230	79.860	71.827
Zprime2_1_2	7.437	7.669	10.341	99.808	103.095	96.598
Zprime2_1_3	8.192	8.197	9.992	73.333	73.132	61.169

S6 Comparison of various approximations to the Gibbs free energy

Table S6: Comparison of Gibbs free energy approximations for selected crystal structures of [emIm][$MeSO_3$]. The names correspond to conventions outlined in e.g. Figure S3. Note that the reference free energy is that of structure labeled "14_EXP" elsewhere, or the energy of the found experimental structure. All numbers are given in $kJ\,mol^{-1}$.

	ΔG^{HA}		ΔG^{QHA}		$\Delta G^{QHA} - \Delta G^{HA}$	
**Name**	**0 K**	**200 K**	**0 K**	**200 K**	**0 K**	**200 K**
14_1	3.684	2.102	5.208	3.536	1.525	1.435
2_2	3.273	1.248	5.305	3.538	2.033	2.291
14_3	2.681	1.178	4.606	3.779	1.925	2.601
14_4	3.203	1.368	5.909	4.221	2.707	2.853
				RMSD	2.091	2.356

Table S7: Comparison of Gibbs free energy approximations for selected crystal structures of [emIm][$EtSO_4$]. The names correspond to conventions outlined in e.g. Figure S6. Note that the reference free energy is that of structure labeled "33_1" elsewhere, or the energy of the lowest $Z' = 1$ structure according to the QHA. All numbers are given in $kJ\,mol^{-1}$.

**Approximation**	ΔG^{HA}		ΔG^{QHA}		$\Delta G^{QHA} - \Delta G^{HA}$	
**Name**	**0 K**	**200 K**	**0 K**	**200 K**	**0 K**	**200 K**
14_2	0.507	0.208	0.444	−0.310	−0.063	−0.518
14_3	0.419	0.447	0.663	0.711	0.244	0.264
14_4	1.097	1.289	0.896	1.270	−0.201	−0.019
14_5	0.904	1.323	1.287	1.523	0.383	0.200
19_6	2.753	2.233	4.288	3.031	1.536	0.798
				RMSD	0.722	0.451

Table S8: Differences between the ΔE values computed on the PBE level of theory and the two used approximations of the Gibbs free energy for crystals of [emIm][$MeSO_3$]. The ΔG values were pulled from Table S6.

		$E_{DFT} - \Delta G$	
**Name**	**PBE**	**HA (0 K)**	**QHA (0 K)**
14_1	3.418	−0.265	−1.790
2_2	3.075	−0.198	−2.231
14_3	2.667	−0.014	−1.939
14_4	3.252	0.049	−2.658
	RMSD	0.167	2.180

Table S9: Differences between the ΔE values computed on the PBE level of theory and the two used approximations of the Gibbs free energy for crystals of [emIm][$EtSO_4$]. The ΔG values were pulled from Table S7.

		$E_{DFT} - \Delta G$	
**Name**	**PBE**	**HA (0 K)**	**QHA (0 K)**
14_2	0.390	−0.117	−0.053
14_3	0.005	−0.414	−0.658
14_4	0.292	−0.805	−0.604
14_5	0.698	−0.206	−0.588
19_6	2.746	−0.006	−1.542
	RMSD	0.418	0.840

S7 Comparison with known experimental data for [emIm][$MeSO_3$]

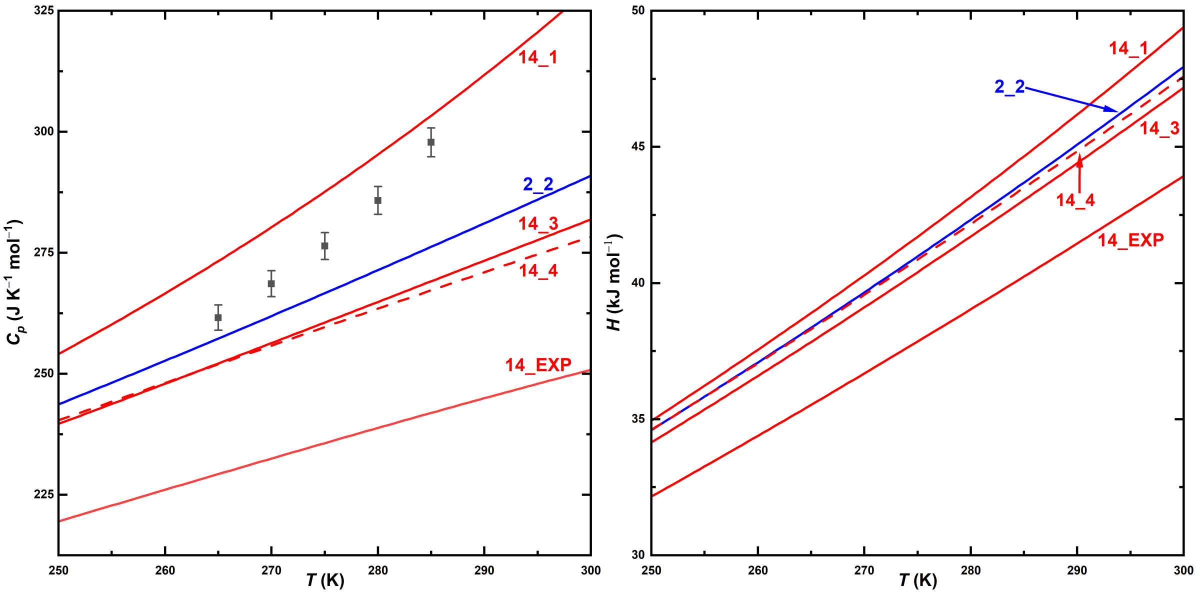

Figure S12: Left: Isobaric heat capacity functions resulting from the quasi-harmonic approximation. The black squares are data for the experimental phase of [emIm][$MeSO_3$] measured in ref.[3] along with their expanded uncertainty (0.95 confidence interval). Right: The integrated heat capacity from the left figure, *i.e.* enthalpy. The different line style for the 14_4 structure is used solely for the purposes of clarity.

Table S10: Comparison between densities of different $Z' = 1$ crystals of [emIm][$MeSO_3$] computed *via* the QHA and the known experimental density of the most stable phase. Note that the uncertainty in temperature determination is given in the paper[3] as the standard $k = 1$, whereas in the densitiy uncertainty is stated as "unrealistically small".

T (K)	Name	Density g cm^{-3}
250	14_EXP	1.342
	14_1	1.261
	2_2	1.301
	14_3	1.311
	14_4	1.287
248 ± 3	reference[3]	1.370

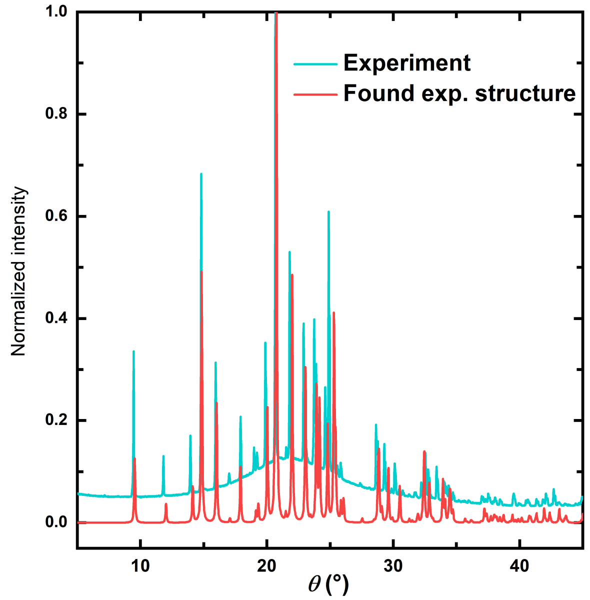

Figure S13: Comparison between the experimental XRPD pattern[3] and the pattern, generated *via* the CCDC Suite, for its computationally discovered counterpart, specifically the PBE optimized structure. Note that the intensities of both spectra were normalized to fit within the scale between 0 and 1.

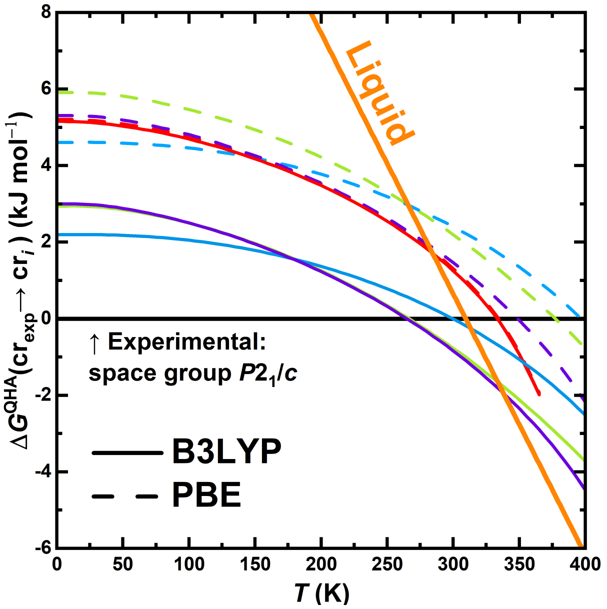

Figure S14: Comparison between the semi-experimental Gibbs energy of the liquid phase of [emIm][$MeSO_3$] and of the Gibbs energies computed for the predicted crystalline phases of [emIm][$MeSO_3$]. The liquid-phase curve was estimated from known experimental data[3] in the following way: Given the experimental enthalpy of fusion and melting-point temperature of the most stable phase, the melting entropy can be established *via* basic thermodynamic relations. Given the Figure presets differences in Gibbs energy, this melting entropy can be thought of as the difference in slope between the liquid curve and the experimental Gibbs energy curve of the found experimental structure. Melting temperatures of individual polymorphs correspond to points where the liquid-phase curve intersects the Gibbs energy profiles of individual polymorphs.

References

[1] M. J. Frisch, G. W. Trucks, H. B. Schlegel, G. E. Scuseria, M. A. Robb, J. R. Cheeseman, G. Scalmani, V. Barone, B. Mennucci, G. A. Petersson, H. Nakatsuji, M. Caricato, X. Li, H. P. Hratchian, A. F. Izmaylov, J. Bloino, G. Zheng, J. L. Sonnenberg, M. Hada, M. Ehara, K. Toyota, R. Fukuda, J. Hasegawa, M. Ishida, T. Nakajima, Y. Honda, O. Kitao, H. Nakai, T. Vreven, J. J. A. Montgomery, J. E. Peralta, F. Ogliaro, M. Bearpark, J. J. Heyd, E. Brothers, K. N. Kudin, V. N. Staroverov, T. Keith, R. Kobayashi, J. Normand, K. Raghavachari, A. Rendell, J. C. Burant, S. S. Iyengar, J. Tomasi, M. Cossi, N. Rega, J. M. Millam, M. Klene, J. E. Knox, J. B. Cross, V. Bakken, C. Adamo, J. Jaramillo, R. Gomperts, R. E. Stratmann, O. Yazyev, A. J. Austin, R. Cammi, C. Pomelli, J. W. Ochterski, R. L. Martin, K. Morokuma, V. G. Zakrzewski, G. A. Voth, P. Salvador, J. J. Dannenberg, S. Dapprich, A. D. Daniels, O. Farkas, J. B. Foresman, J. V. Ortiz, J. Cioslowski and D. J. Fox, *Gaussian 16*, 2016, `https://gaussian.com/`.

[2] C. R. Groom, I. J. Bruno, M. P. Lightfoot and S. C. Ward, The Cambridge Structural Database, *Acta Crystallographica Section B Structural Science, Crystal Engineering and Materials*, 2016, **72**, 171–179.

[3] V. Štejfa, J. Rohlíček and C. Červinka, Phase behaviour and heat capacities of selected 1-ethyl-3-methylimidazolium-based ionic liquids II, *The Journal of Chemical Thermodynamics*, 2021, **160**, 106392.
